# Metformin-induced ablation of microRNA 21-5p releases Sestrin-1 and CAB39L antitumoral activities

**DOI:** 10.1038/celldisc.2017.22

**Published:** 2017-07-04

**Authors:** Claudio Pulito, Federica Mori, Andrea Sacconi, Frauke Goeman, Maria Ferraiuolo, Patrizia Pasanisi, Carlo Campagnoli, Franco Berrino, Maurizio Fanciulli, Rebecca J Ford, Massimo Levrero, Natalia Pediconi, Ludovica Ciuffreda, Michele Milella, Gregory R Steinberg, Mario Cioce, Paola Muti, Sabrina Strano, Giovanni Blandino

**Affiliations:** 1 Molecular Chemoprevention Unit, Italian National Cancer Institute ‘Regina Elena’, Rome, Italy; 2 Oncogenomic and Epigenetic Unit, Italian National Cancer Institute ‘Regina Elena’, Rome, Italy; 3 Department of Preventive & Predictive Medicine, Fondazione IRCCS Istituto Nazionale Dei Tumori, Milan, Italy; 4 Unit of Endocrinological Gynecology, Ospedale Sant’Anna di Torino, Turin, Italy; 5 SAFU, ‘Regina Elena’ National Cancer Institute, Rome, Italy; 6 Division of Endocrinology and Metabolism, Department of Medicine, McMaster University, Hamilton, Ontario, Canada; 7 Epigénétique et Épigénomique des Carcinomes Hépathocellulaires Viro-Induits du Centre de Recherche en Cancérologie de Lyon, Lyon, France; 8 Department of Molecular Medicine, Sapienza University of Rome, Rome, Italy; 9 Division of Medical Oncology A, Italian National Cancer Institute ‘Regina Elena’, Rome, Italy; 10 Department of Oncology, Juravinski Cancer Center, McMaster University, Hamilton, Ontario, Canada

**Keywords:** cancer metabolism, everolimus, metformin, miRNA, mTOR

## Abstract

Metformin is a commonly prescribed type II diabetes medication that exhibits promising anticancer effects. Recently, these effects were found to be associated, at least in part, with a modulation of microRNA expression. However, the mechanisms by which single modulated microRNAs mediate the anticancer effects of metformin are not entirely clear and knowledge of such a process could be vital to maximize the potential therapeutic benefits of this safe and well-tolerated therapy. Our analysis here revealed that the expression of miR-21-5p was downregulated in multiple breast cancer cell lines treated with pharmacologically relevant doses of metformin. Interestingly, the inhibition of miR-21-5p following metformin treatment was also observed in mouse breast cancer xenografts and in sera from 96 breast cancer patients. This modulation occurred at the levels of both pri-miR-21 and pre-miR-21, suggesting transcriptional modulation. Antagomir-mediated ablation of miR-21-5p phenocopied the effects of metformin on both the clonogenicity and migration of the treated cells, while ectopic expression of miR-21-5p had the opposite effect. Mechanistically, this reduction in miR-21-5p enhanced the expression of critical upstream activators of the AMP-activated protein kinase, calcium-binding protein 39-like and Sestrin-1, leading to AMP-activated protein kinase activation and inhibition of mammalian target of rapamycin signaling. Importantly, these effects of metformin were synergistic with those of everolimus, a clinically relevant mammalian target of rapamycin inhibitor, and were independent of the phosphatase and tensin homolog status. This highlights the potential relevance of metformin in combinatorial settings for the treatment of breast cancer.

## Introduction

The ability to suppress hepatic gluconeogenesis and reduce blood glucose levels, together with its safe profile have made the dimethyl-biguanide metformin a worldwide prescribed agent for type II diabetes patients[1]. Recent epidemiological studies supported a new therapeutic role for metformin toward the prevention and treatment of cancer. In detail, type II diabetes patients treated with metformin showed an important reduction in cancer risk compared with those treated with other hypoglycemic therapies[2]. Chronic treatment with metformin in diabetic patients was associated with a reduction, in several types of cancer, of the incidence compared with other antidiabetic agents[3], findings which led to the initiation of many clinical trials testing the preventive effects of the drug in numerous cancers. Given its excellent safety profile, metformin has also been used to enhance the effects of chemotherapies[4, 5]. Further, mammalian target of rapamycin (mTOR) inhibition, mediated by metformin, sensitized breast cancer cell lines and thyroid cancer cells to cytotoxic effect of everolimus and vemurafenib, respectively[6, 7]. Despite these promising clinical data, the mechanisms by which metformin elicits antiproliferative effects alone or in combination with other agents are not fully understood but vital to understand in order to guide clinical development. The AMP-activated-protein-kinase (AMPK) is a main mediator of the metformin-anticancer activities. It is activated upon phosphorylation of Thr^172^ within the catalytic alpha-subunit by the LKB1–STRAD–CAB39L (CAB39L is also known as MO25β) complex[8–10]. The calcium-binding protein 39 (Cab39 or MO25) exists as two isoforms, encoded by the closely related genes MO25α (CAB39) and MO25β (CAB39L). It is a scaffold protein that binds and stabilizes the LKB1 activation loop in a conformation required for phosphorylation of substrates[8]. In particular, its presence enhances the regulatory effect of the pseudokinases, STE20-like kinase family, STRADα and STRADβ, on the activity of LKB1. AMPK is further activated through Sestrin-1 (SESN1)[11], this latter being a critical factor at the interface between metabolism and cancer[12, 13]. Importantly, in cells lacking LKB1 or AMPK cell growth is uncontrolled[14], as being at least partially fuelled by a glycolytic switch that maximizes macromolecule synthesis. Those effects were counteracted by metformin treatment[15, 16]. Moreover, AMPK activation inhibited the mTOR pathways, which is constitutively activated in a broad spectrum of tumors[17], thus representing an appealing target for cancer treatment. As a consequence, mTOR was no longer able to promote protein synthesis pathways through the activation of S6 kinase (S6K) and translation initiation factor 4E binding protein 1 (4EBP1). This resulted in an inhibition of the mTOR oncogenic signaling, which mainly required S6K and active protein synthesis[18]. Surprisingly, despite the well-known actions of metformin toward AMPK, the mechanisms by which metformin elicits activation of the kinase in tumors are still not fully understood. Notably, in contrast to the liver which has high concentrations of metformin that disrupt oxidative phosphorylation, the tumor concentrations of metformin are much lower. However, such doses were found to be sufficient to activate AMPK in tumor xenografts[19–21].

We recently showed that metformin-induced anticancer effects correlated with decreased levels of multiple microRNAs (miRNA), through an E2F1/3-dependent modulation of the DICER processing enzyme. This elicited metabolic reprogramming *in vivo* and *in vitro* and correlated with metformin’s anticancer effects[22]. miRNAs are important modulators of the cancer phenotype. It has been demonstrated that an aberrant expression of a single miRNA can, alone, induce and sustain cancer, while its silencing could induce reversion of the tumor[23–25]. Within this line, miR-21-5p represents a paradigmatic example. Its inactivation has been found to induce a complete tumor regression, thus demonstrating that cancer can be addicted to a single oncomiR[26]. miR-21-5p was found, also, to be overexpressed in most of the tumor types examined so far, including neuroblastoma, glioblastoma, colorectal, lung, breast, pancreas, head and neck, gastric cancer, leukemia and lymphoma[27–32]. Moreover, in a large-scale analysis of 540 human samples representing 6 different tumor histotypes, miR-21 was the only upregulated miRNA common to all the tumor specimens[33]. Further, its circulating levels in serum or plasma were found to be diagnostic and prognostic for detecting and staging breast[34], pancreatic[35] and colorectal cancer[36].

In the current study, we have discovered that metformin induced a decrease in the oncogenic miR-21-5p in cultured human breast cancer cells *in vitro*, in mouse xenografts and in sera of breast cancer patients. Decreased miR-21-5p released the expression of two, yet undiscovered miR-21-5p targets, SESN1 and CAB39L, leading to the activation of AMPK. This caused inhibition of mTOR following the activation of the AMPK cascade and strictly correlated with a reduced clonogenicity, migration and invasion of multiple breast cancer cell lines. Changes in the miR-21-5p levels and of the stoichiometry of the CAB39L-LKB1 complexes were important for metformin’s effects in both PTENwt and PTENdel cell lines. Finally, we explored the possibility of combining metformin with everolimus to enhance the activity of the latter, which was weaker in breast cancer cell lines harboring a PTENwt allele. Thus here we further characterized the complex yet promising action of metformin and indicated a potential, translationally relevant combination therapy.

## Results

### Metformin modulates the levels of miR-21-5p

**Figure 1:**
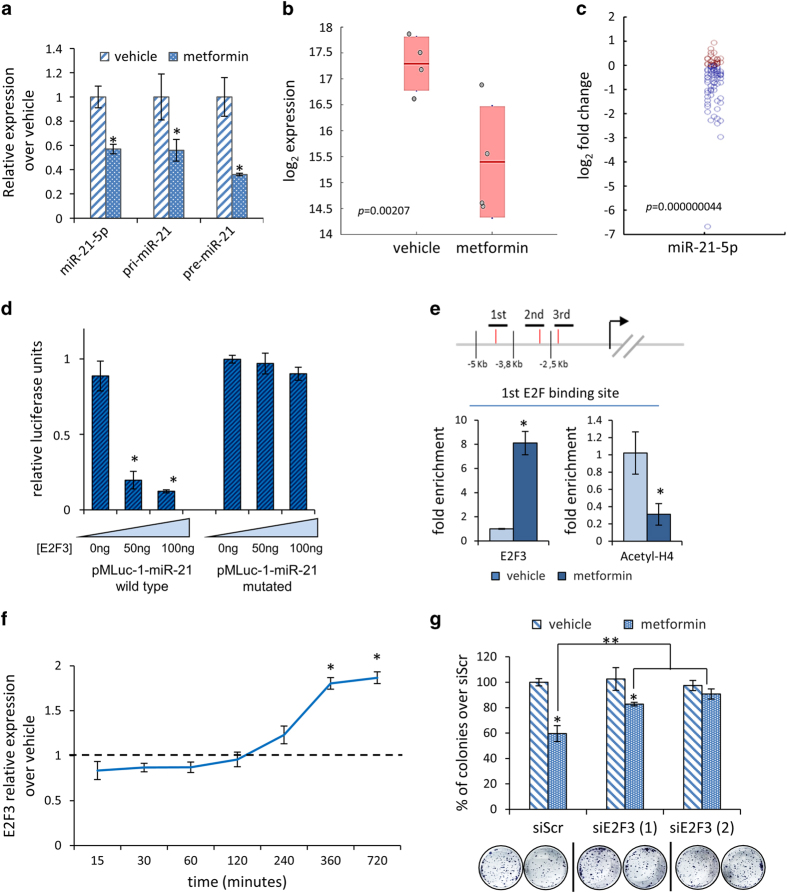
Metformin downregulated the miR-21-5p expression. (**a**) Quantitative PCR. Expression of the pri- and pre-miR-21-5p in SUM159PT cells treated with metformin (0.5 mM) for 24 h. Bars indicate the average of three independent experiments. Statistics (*t*-test): *P*<0.05. (**b**, **c**) Modulation of miR-21-5p happened *in vivo*. (**b**) Relative levels of miR-21-5p (log2) expression in pooled SUM159PT tumors treated intraperitoneally with vehicle (*n*=4) or metformin (*n*=4) (16 mg kg^−1^, bi-weekly, for 10 weeks) when the average starting tumor volume was 0.2 cm^3^. Statistics (*t*-test): *P*<0.05. (**c**) Scatter plot indicating the miR-21-5p fold changes (log2) in the sera of women (*n*=96) with established breast cancer collected before and after 3 months of daily treatment with metformin (500 mg day^−1^). Each circle indicates a patient with decreased (blue ones) or increased (red ones) levels of miR-21-5p as compared with the pretreatment levels. Statistics: (Wilcoxon sign-rank test, matched samples) *P*<0.001. (**d**) Luciferase assay. HEK-293 cells co-transfected with the wild-type or mutated miR-21-5p promoter/LUC reporter construct and with different doses of E2F3. Histograms show the normalized luciferase values from three independent experiments. Statistics (*t*-test): *P*<0.05. (**e**) E2F3 binding to the miR-21-5p promoter mediated the effect of metformin. Upper panel. Predicted E2F-binding sites in the miR-21-5p promoter. Horizontal bars indicate the three different E2F-binding sites amplified in the chromatin immunoprecipitation experiment. Lower panel. Histograms showing the amplification of the E2F-binding sites in the chromatin precipitated with anti-E2F3 and anti-acetylated Histone H4 (Acetyl-H4), from vehicle and metformin-treated (0.5 mM, 24 h) SUM159PT cells. Statistics (*t*-test): *P*<0.05. Please see additional data in Supplementary Figures S1d and e (**f**) Metformin induced E2F3 transcriptional expression. Quantitative PCR. Relative expression of E2F3 in SUM159PT cells treated with metformin (0.5 mM) for different times (0–720 min). Statistics (*t*-test): *P*<0.05 over untreated cell (dashed line). (**g**) Altering the levels of E2F3 impaired the metformin effect on the SUM159PT clonogenic ability. Histograms showing the average percentage of colonies formed by SUM159PT cells transfected with two different E2F3 siRNAs (or scrambled control) and treated with vehicle or metformin (0.5 mM, 24 h) before seeding at clonal density. Statistics (*t*-test): *P*<0.05 *Significant versus vehicle, **Significant versus control transfected cells treated with metformin.

We have shown that metformin anticancer effects include and require a widespread modulation of miRNAs[22]. Here we deepened such observation and focused on miR-21-5p, among the miRNAs prominently modulated in the SUM159PT triple-negative breast cancer cells, treated with pharmacologically relevant doses of metformin (0.5 mM) (Figure 1a and Supplementary Figure S1a)[21, 37]. By using quantitative PCR (qPCR), we specifically evaluated the levels of the miR-21-5p precursors (pre-miR-21-5p and pri-miR-21-5p) in the treated SUM159PT cells (Figure 1a). This revealed that both the miR-21-5p species were steadily downregulated, as compared with the control cells (*P*<0.05), thus suggesting that modulation of the miR-21-5p levels by metformin may occur transcriptionally. This phenomenon was common to three additional breast cancer cell lines (MCF-7, BT-474, BT-549) (Supplementary Figure S1b). Next, we assessed whether the modulation of miR-21-5p by metformin may take place in more clinically relevant experimental systems (Figure 1b and c). First, we evaluated the miR-21-5p levels in RNA extracted from pooled SUM159PT tumors of mice treated with vehicle or metformin (16 mg kg^−1^, bi-weekly), for 10 weeks, after the average starting tumor volume was 0.2 cm^3^. This showed that the levels of miR-21-5p significantly dropped in the metformin-treated mice, as compared with the vehicle-treated ones (Figure 1b). To validate these experimental data, we analyzed the levels of miR-21-5p in the sera of patients (*n*=96) with history of breast cancer, collected before and after 3 months of daily treatment with metformin (500 mg day^−1^)[38]. We observed that serum levels of the miR-21-5p were decreased in most of the patients after a 3-month treatment (Figure 1c). Additionally, we found that transfection of SUM159PT and of three more breast cancer cell lines (MCF-7, BT-474, BT-549) with a miR-21-5p mimic or inhibitor strongly recapitulated the effect of metformin treatment on both the clonogenicity and migration of the treated cells (Supplementary Figure S1f–i). Thus downregulation of miR-21-5p by metformin happened *in vitro* and *in vivo* and affected clonogenicity and migration of four independent breast cancer cell lines. Intriguingly, *in silico* analysis of the miR-21-5p promoter revealed the presence of three E2F-binding sites, respectively, at −4530/−4515 (first site), −2834/−2814 (second site) and −2216/−2204 (third side) base pairs from translational starting sites (ATG) (Figure 1e). E2F3 was capable of transcriptional repression[39] and was already shown to be a target of metformin in a similar experimental context[22, 40]. Thus we evaluated whether metformin treatment may cause variations in the occupancy of the miR-21-5p promoter by E2F3. First, we verified that, *in vitro*, transfection of increasing doses of E2F3 strongly reduced the activity of a miR-21-5p-promoter-LUC vector containing the proximal E2F3-binding site (Figure 1d). Conversely, the repressive effect of E2F3 was lost when its binding consensus sequence was mutated, ensuring specificity of our observation (Figure 1d). Next, chromatin immunoprecipitation revealed increased binding of E2F3 at all the three sites of the miR-21-5p promoter analyzed (Figure 1e and Supplementary Figure S1d and e). This correlated with decreased signal, in the same regions, for the hyper-acetylated Histone H4, which reached statistical significance in two out of the three regions (Figure 1e and Supplementary Figure S1d and e). The presence of hyper-acetylated Histone H4 is generally considered an indicator of transcriptionally active chromatin[41]. Thus its decrease, concomitant with increased binding of E2F3, indicated transcriptional repression and correlated with decreased activity of the miR-21-5p promoter. Moreover, qPCR showed an increase of the E2F3 transcript in time after metformin treatment (Figure 1f), which paired the increased presence of this factor on the miR-21-5p promoter (Figure 1e). Finally, small interfering RNA (siRNA)-mediated silencing of E2F3 impaired the ability of metformin at reducing the colony-forming ability of treated SUM159PT cells (Figure 1g). This caused the levels of miRNA 21-5p being left unstimulated in the presence of metformin (Supplementary Figure S1c).

### CAB39L and SESN1 are modulated by metformin-miR-21-5p

**Figure 2:**
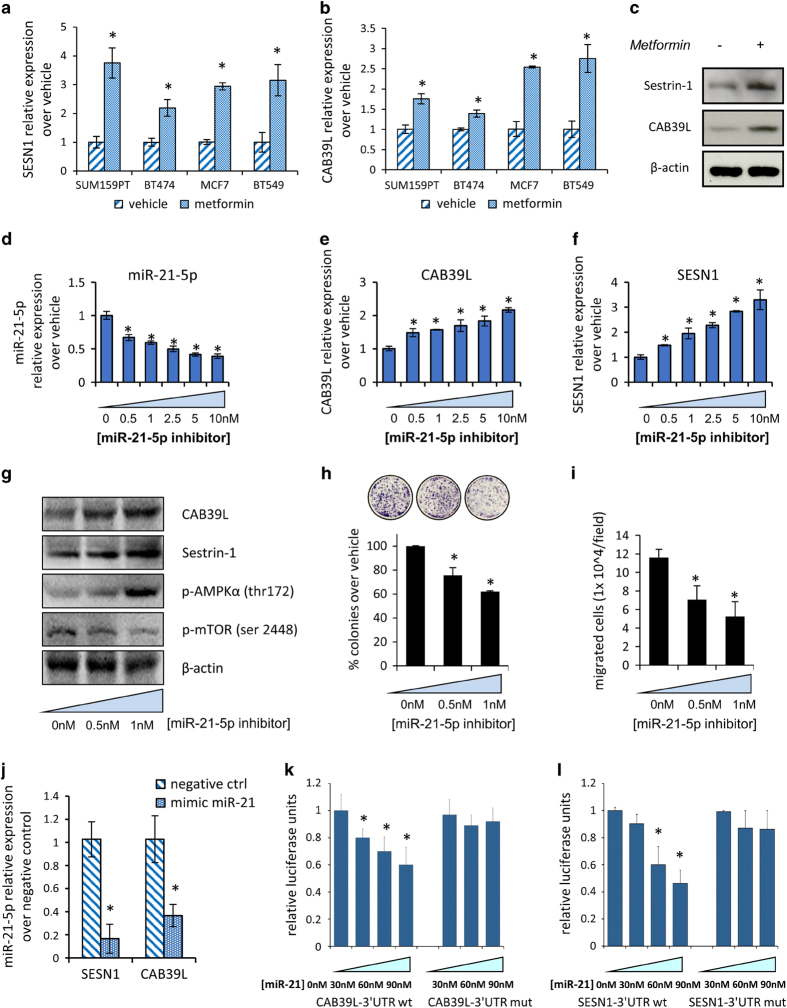
Calcium-binding protein 39-like (CAB39L) and Sestrin-1 (SESN1) are targets of metformin-miR-21-5p. (**a**, **b**) Quantitative PCR. Histograms showing the relative levels of CAB39L (**a**) and SESN1 (**b**) mRNAs in SUM159PT, BT-474, MCF-7 and BT-549 cells, treated with vehicle or metformin (0.5 mM) for 24 h. Statistics (*t*-test): *P*<0.05. (**c**) Representative western blotting with anti-CAB39L or SESN1 antibodies of whole-cell lysates from SUM159PT cells treated with vehicle or metformin (0.5 mM) for 24 h. Actin staining was used as a loading control. (**d**–**i**) Modulation of CAB39L and SESN1 by metformin required miR-21-5p. (**d**–**f**) Quantitative PCR. Histograms showing the relative levels of miR-21-5p (**d**), CAB39L (**e**) and SESN1 (**f**) mRNAs in SUM159PT transfected with different doses of miR-21-5p inhibitor. Statistics (*t*-test): *P*<0.05. (**g**) Representative western blotting of whole-cell lysates from SUM159PT cells treated as from panel (**d**–**f**), stained with the indicated antibodies. Actin staining was used as a loading control. (**h**, **i**) Colony-forming assay (**h**) and invasion assay (**i**). Histograms showing average colony counts (**h**) or the number of migrated cells (**i**) of SUM159PT cells treated as from panel (**g**). Statistics (*t*-test): *P*<0.05. (**j**) Quantitative PCR. mRNA levels of SESN1 or CAB39L in SUM159PT cells expressing either a control vector or a miR-21-5p mimic vector. Bars indicate the average of three independent experiments. Statistics (*t*-test): *P*<0.05. (**k**, **l**) Dual luciferase assay. SUM159PT cells co-transfected with the wild-type or mutated CAB39L (**k**) or SESN1 (**l**) 3′-untranslated region luciferase reporter and, subsequently, with either a scrambled- or a specific miR-21-5p agonist. Histograms show the normalized luciferase values from three independent experiments. Statistics (*t*-test): *P*<0.05.

To study the mechanistic details of the metformin effect on miR-21-5p, we performed an extensive *in silico* analysis to search for mRNAs that contained miR-21-5p binding sites in their 3′ untranslated region (UTR) and whose levels anticorrelated with those of the miR-21-5p. We found that both CAB39L and SESN1 mRNAs fulfilled those two criteria and this happened similarly in gastric cancer, head and neck and breast cancer cohorts of patients (Table 1). First, RNA-Seq of metformin-treated SUM159PT cells confirmed that SESN1 and CAB39L transcripts were, indeed, significantly upregulated when compared with control-treated cells (Supplementary Figure S2a). We confirmed such a similar modulation in three additional breast cancer cell lines (MCF-7, BT-474, BT-549) by qPCR (Figure 2a and b). Finally, increased mRNA levels paired with increased protein levels of both SESN1 and CAB39L in the treated SUM159PT cells (Figure 2c). We also found increased CAB39L expression in sections of tumors excised from SUM159PT xenografted mice treated with metformin (Supplementary Figure S2b). On a similar note, Kaplan–Meier analysis showed that breast cancer patients with higher expression of both SESN1 and CAB39L exhibited better survival (Supplementary Figure S2c). Altogether, these data suggested a possible clinically relevant effect of metformin on the two miR-21-5p targets. To validate these findings, we studied the effect of exogenously added miR-21-5p inhibitor on the levels of CAB39L and SESN1 (Figure 2d–f). We found increased mRNA and protein levels of CAB39L and SESN1 (Figure 2e–g) in cells transfected with 0.5–1 nM of miR-21-5p inhibitor, the latter concentrations of antagonist being those that more closely mimicked the effect of metformin on the endogenous mRNAs. This correlated with a significant reduction in the ability of the interfered cells at forming colonies and to migrate (Figure 2h and i). Conversely, we found that a miR-21-5p mimic transfected in SUM159PT cells caused a decrease of both SESN1 and CAB39L endogenous mRNAs (Figure 2j) and reduced, dose-dependently, both SESN1 3′-UTR- and CAB39L 3′-UTR-driven luciferase activity (Figure 2k and l). Such effect was not observed when the miR-21-5p seed sequence was mutated, ensuring the specificity of our observation (Figure 2k and l). Thus the miR-21-5p could directly modulate the SESN1 and CAB39L levels, thereby strictly mimicking the effect of metformin on those mRNAs.

### Biochemical effects of metformin-induced increase of CAB39L and SESN1

**Figure 3:**
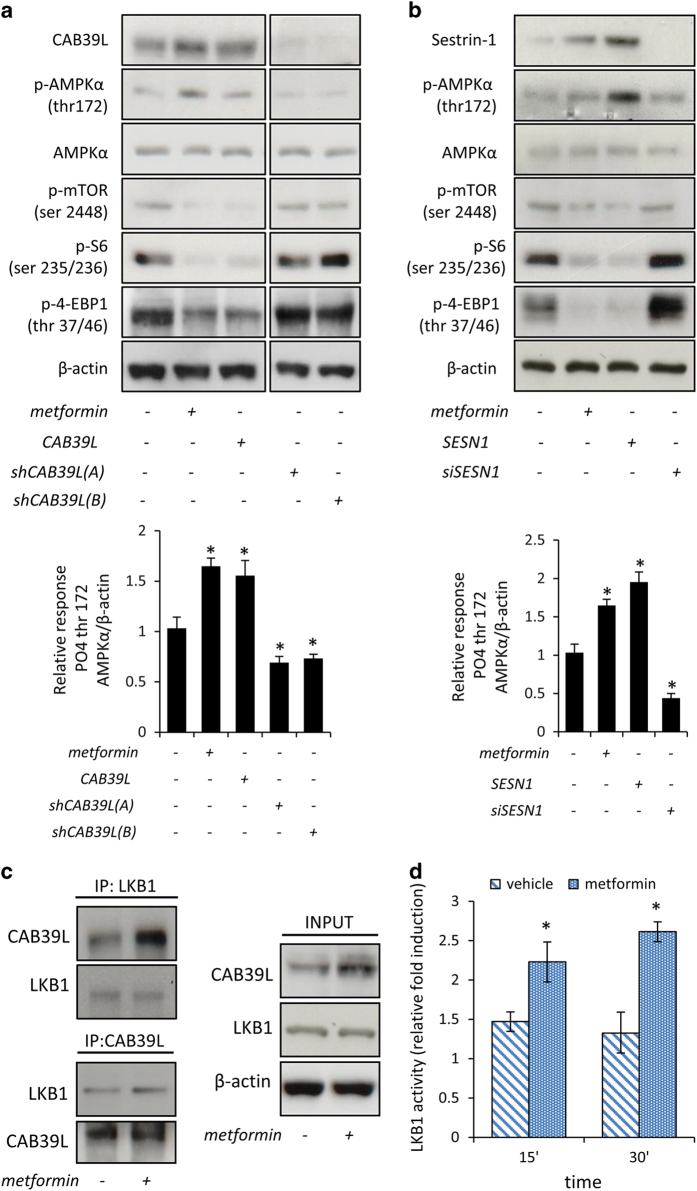
Calcium-binding protein 39-like (CAB39L) and Sestrin-1 (SESN1) mediated the anticancer effects of metformin through AMP-activated protein kinase–mammalian target of rapamycin (AMPK-mTOR) signaling. (**a**, **b**) Altering the levels of CAB39L and SESN1 mimicked the effect of metformin on the AMPK/mTOR signaling axis. (**a**) Upper panel. Representative western blotting of whole-cell lysates from SUM159PT treated with vehicle or metformin or transfected with the expression vectors coding for CAB39L or for two shRNAs against CAB39L, stained with the indicated pan- and phospho-specific antibodies. Lower panel. Quantitative densitometry of phospho-thr^172^ AMPKα calculated from the analysis of three western blottings including that in panel (**a**). (**b**) Upper panel. Representative western blotting of similarly stained whole-cell lysates from of the same cells treated as from panel (**a**) and transfected with the expression vectors for SESN1 or with a siRNA against SESN1. Lower panel. Quantitative densitometry of phospho-thr^172^ AMPKα calculated from the analysis of three western blottings including that in panel (**b**). (**c**) The LKB1–CAB39L complex was modulated by metformin. Representative western blotting of whole-cell lysates of vehicle- and metformin-treated SUM159PT cells, immunoprecipitated with anti-LKB1 and anti-CAB39L antibodies (upper left and lower left panels, respectively). Actin staining was used as a loading control for the input material (right panel). (**d**) Metformin stimulates LKB1 kinase activity. Immuno-kinase assay. Histograms showing the enzymatic activity of LKB1: immunoprecipitates from HEK-293 cells treated or not with 0.5 mM of metformin for 24 h. Mean of two independent experiments. Statistics (*t*-test): *P*<0.001.

Metformin is an AMPK activator and we assessed whether the effect of altering CAB39L and SESN1 levels in metformin-treated cells would impinge on the AMPK signaling (Figure 3a and b). Staining of whole-cell lysates obtained from vehicle- and metformin-treated cells revealed that metformin-induced levels of CAB39L and SESN1 in SUM159PT cells tightly matched the activation of AMPK and the downregulation of the mTOR signaling, as assessed by reduced (phospho-ser^2448^)-mTOR, (phospho-ser^235/236^)-S6 and (phospho-thr^37/46^)-4-EBP1 (Figure 3a and b, upper and lower panels, respectively). siRNA-mediated downregulation of CAB39L and SESN1 exerted opposite effects to those of metformin on the phosphorylation of the mentioned signaling molecules (Figure 3a and b, upper and lower panels, respectively). To further characterize, mechanistically, this signaling, we found that depletion of either CAB39L or SESN1 impinged on the AMPK activation and on the mTOR inhibition triggered by metformin, which were reduced and increased, respectively, in transfected HEK-293 cells (Supplementary Figure S3a and b). On the contrary, cells transfected with both the coding sequence of CAB39L and SESN1 showed higher levels of phospho-AMPK on residue thr^172^ and a significant downregulation of the phospho-mTOR on the residue ser^2448^ protein levels, as compared with their control (Supplementary Figure S3c). Further, we found that exogenous increase of the miR-21-5p could counteract the effects of metformin, thus causing reduced AMPK activation and increased phospho-mTOR signal, as compared with the metformin-treated, control-transfected cells (Supplementary Figure S3d, upper and lower panels, respectively). On the other hand, the same cells transfected with a miR-21-5p agonist, in the presence of SESN1 and CAB39L overexpression and without metformin treatment, exhibited AMPK activation and mTOR inhibition (Supplementary Figure S3d). Altogether, these experiments revealed a rather complex interplay between miR-21-5p and its two targets in metformin-treated breast cancer cells.

Mechanistically, CAB39L is a scaffold protein that binds to LKB1, thereby favoring the downstream activation of AMPK[42]. Thus we assessed whether metformin treatment enhanced the formation of a LKB1/CAB39L protein complex. Reciprocal coprecipitation experiments revealed that the LKB1/CAB39L complex was more evident upon metformin treatment, at least partially due to the increased of CAB39L levels (Figure 3c, left and right, respectively). This correlated with the observed higher phosphorylation of AMPK (Figure 3a and b). Finally, we performed an LKB1-immune kinase assay and found that metformin increased LKB1 kinase activity as assessed by using a specific substrate peptide, (Figure 3d). In line with this, we found that downregulation of LKB1 by siRNA impaired the effect of metformin on the clonogenicity of the transfected cells (Supplementary Figure S3e).

### Modulation of CAB39L and SESN1 impinged on clonogenicity and invasiveness of breast cancer cells

**Figure 4:**
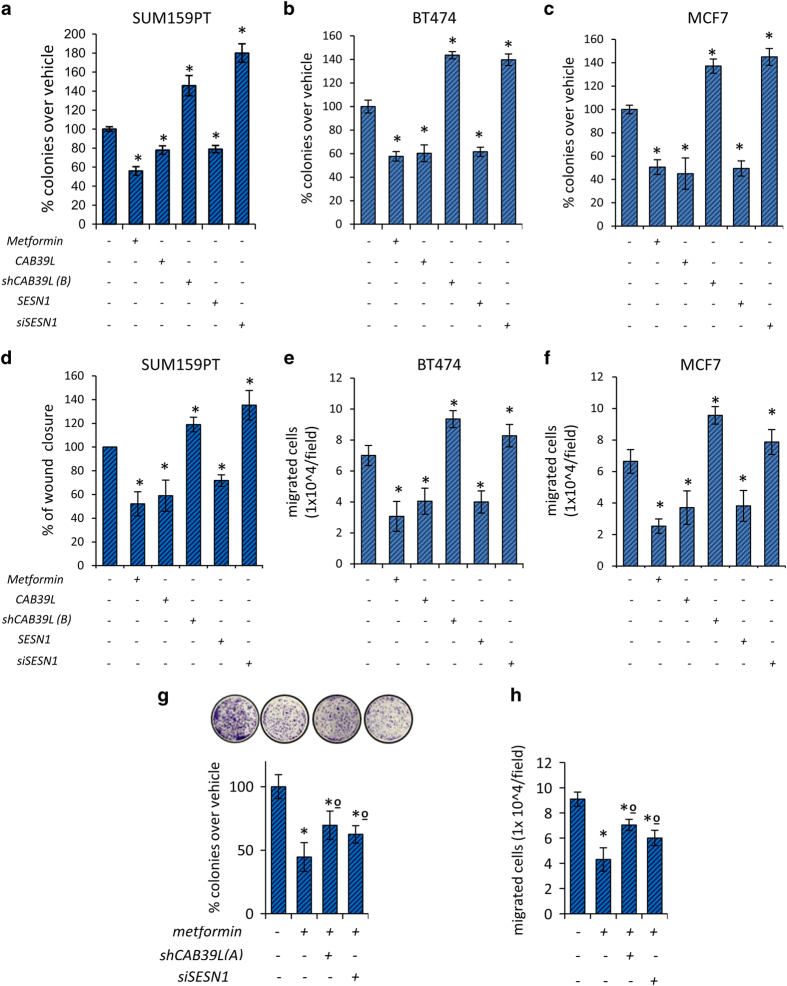
Modulation of calcium-binding protein 39-like (CAB39L) and Sestrin-1 (SESN1) underlies metformin anticancer activities. (**a**–**c**) Colony-forming assay. Histograms showing average colony counts of SUM159PT (**a**), BT-474 (**b**) and MCF-7 (**c**) cells expressing either a control vector or a CAB39L-expressing vector or CAB39L- or SESN1-targeting shRNAs and treated with vehicle or metformin (0.5 mM) before seeding at clonal density. Statistics (*t*-test): *P*<0.05. (**d**) Wound healing assay. Percentage of wound closure (over vehicle) of SUM159PT cells transfected and treated as from panel (**a**–**c**), for 48 h. Statistics (*t*-test): *P*<0.05. (**e**, **f**) Invasion assay. BT-474 (**e**) and MCF-7 (**f**) cells were treated with vehicle or metformin (0.5 mM) for 24 h and the number of migrated cells was scored. (**g**, **h**) Colony-forming assay (**g**) and invasion assay (**h**). Histograms showing average colony counts (**g**) or the number of migrated cells (**h**) of SUM159PT cells transfected either with a control vector or CAB39L- or SESN1-targeting shRNAs and treated with vehicle or metformin (0.5 mM). Histograms indicating the average±s.e. of triplicate experiments. Statistics (*t*-test): *P*<0.05. *Significant versus vehicle, ºSignificant versus untransfected, metformin-treated ones.

We evaluated the biological consequences of metformin–miR-21-5p modulation of CAB39L and SESN1 by means of clonogenic and migration assays (Figure 4g–h and Supplementary Figure S4a–f). Metformin treatment reduced the clonogenicity of SUM159PT and that of the BT-474 and MCF-7 cells (Figure 4a–c) and affected their migration as well (Figure 4d–f, Supplementary Figure S4a and b). This occurred similarly for metformin-treated BT-549 cells (Supplementary Figure S4c and d). The overexpression of either CAB39L or SESN1 mimicked the effect of metformin, while the depletion of CAB39L or SESN1 increased both clonogenicity and migration of all the breast cancer cell lines tested (Figure 4a–f, Supplementary Figure S4a–d). This suggested that the anticancer effect of metformin could at least partially be mediated by these two novel miR-21-5p targets. On the other hand, downregulation of either SESN1 or CAB39L impaired, significantly (*P*<0.05), the ability of metformin to affect colony number and migration (Figure 4g and h). These data suggest that CAB39L or SESN1 depletion counteracted the metformin antitumorigenic effects. In line with these observations, we also found that, in SUM159PT transfected with a miR-21-5p agonist, the effect of metformin was attenuated, again in terms of colony-forming assay and migration assays (Supplementary Figure S4e and f). Finally, we evaluated the effect of overexpressing miR-21-5p, CAB39L and SESN1 in SUM159PT. The triple-transfected cells were significantly less able at forming colonies and to migrate when compared with the untreated ones and their behavior was similar to the metformin-treated ones (Supplementary Figure S4e and f). It is worthy to note that both CAB39L and SESN1 constructs do not have 3′-UTR, and consequently miR-21-5p could not affect their translation. Thus CAB39L and SESN1 overexpression mimicked metformin effects.

### Phosphatase and tensin homolog (PTEN) was not involved in the metformin-mediated anticancer effect

**Figure 5:**
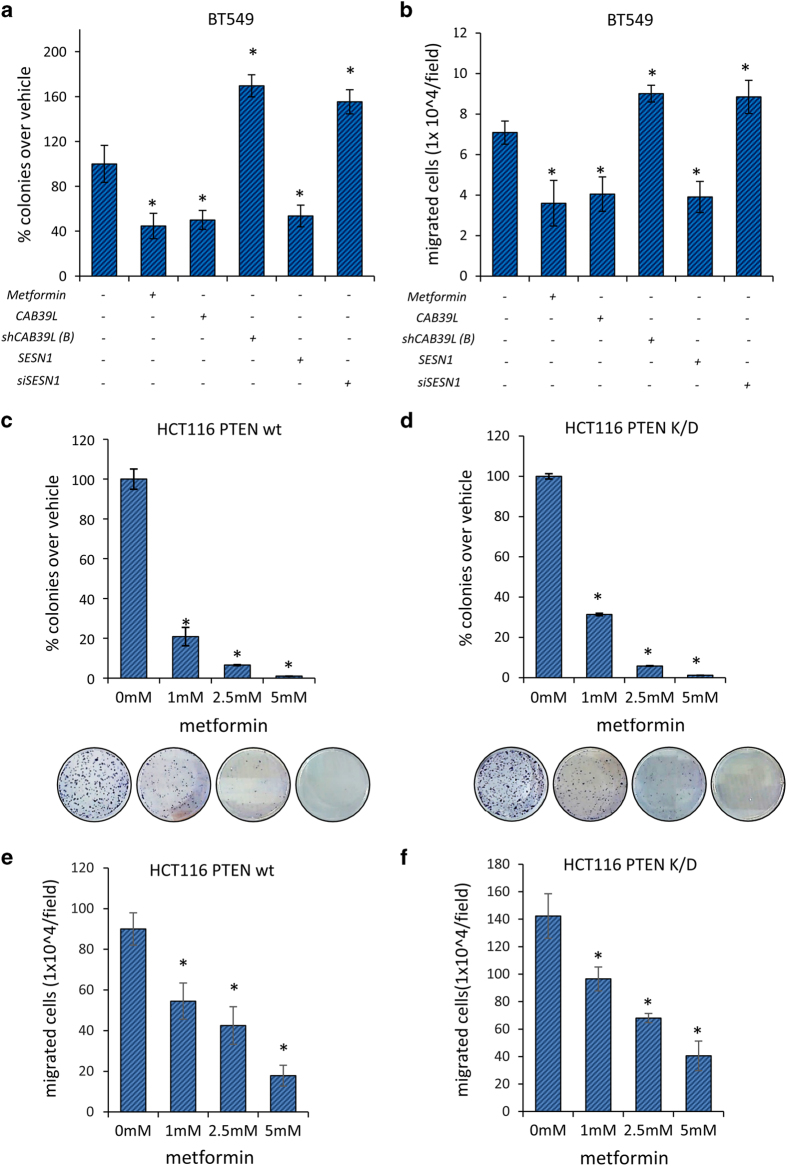
Metformin effects were not influenced by the status of phosphatase and tensin homolog (PTEN). (**a**) Colony-forming assay. Histograms showing average colony counts of BT-549 cells expressing either a control vector or a calcium-binding protein 39-like (CAB39L)-expressing vector or CAB39L- or Sestrin-1 (SESN1)-targeting shRNAs or and treated with vehicle or metformin (0.5 mM) before seeding at clonal density. Statistics (*t*-test): *P*<0.05. (**b**) Invasion assay. BT-549 cells transfected and treated as from panel (**a**) for 24 h and the number of migrated cells was scored. Statistics (*t*-test): *P*<0.05. (**c**, **d**) Clonogenic assay. Isogenic HCT116 bearing a wt PTEN (**c**) or a shRNA targeting PTEN (PTEN K/D) (**d**) were treated with vehicle or metformin (0.5 mM) and colonies stained and counted 8 days later. Histograms indicating the average±s.e. Statistics (*t*-test): *P*<0.05. (**e**, **f**) Invasion assay. The cells were treated with vehicle or metformin (0.5 mM) for 24 h and the percentage of migrated cells was scored. Histograms indicating the average±s.e. of triplicate experiments. Statistics (*t*-test): *P*<0.05.

The tumor-suppressor PTEN is a well-known target of the miR-21-5p[43], and thus we aimed at addressing whether modulation of PTEN may influence our conclusion in this experimental system. First, we found that metformin treatment reduced colony formation and migration of the PTEN-devoid BT-549 cells (Figure 5a and b, respectively). However, as being a long-grown cell line, the BT-549 cells may harbor additional genetic lesions, in addition to PTEN deletion, ultimately influencing the interpretation of the results. Thus we evaluated the effect of metformin on matched isogenic HCT116 cells, virtually differing each other only for the deletion of the PTEN gene. This revealed that metformin treatment affected similarly both the clonogenicity and migration of the isogenic colon carcinoma cells in a dose-dependent way (Figure 5c and d and e and f, respectively). Thus metformin effect may appear to be independent of the PTEN status.

### Metformin synergized with the mTOR inhibitor everolimus

**Figure 6:**
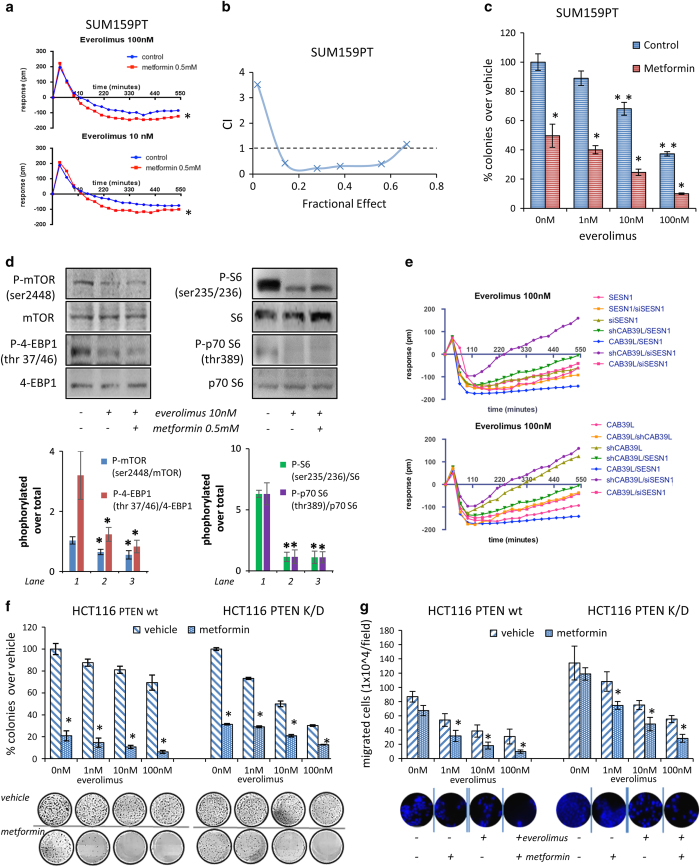
Metformin synergized with everolimus. (**a**) Graphs indicating the change in impedance of SUM159PTcells pretreated or not with metformin (0.5 mM) for 24 h and subsequently treated with everolimus (10–100 nM) for 0–550 min to evaluate early changes in cell fitness. Statistics (*t*-test): *P*<0.05 (versus control). (**b**) Combination index (CI) versus the fractional effect obtained from SUM159PT cells treated simultaneously with metformin (0.5 mM) and everolimus (0-100 nM) for 72 h. (**c**) Histograms showing the average percentage of colonies formed by SUM159PT cells pretreated with vehicle or metformin (0.5 mM) and subsequently treated with everolimus (0–100 nM). Bars indicate the average±s.e. of triplicate experiments. Statistics (*t*-test): *P*<0.05. (**d**) Everolimus and metformin treatment synergistically affected mammalian target of rapamycin (mTOR) signaling. Representative western blotting (upper panel) and quantitative densitometry (lower panel) phospho-ser^2448^-mTOR, (phospho-ser^235/236^)-S6, (phospho-thr^37/46^)-4-EBP1 and (phospho-thr^389^)-p70 S6 normalized to their respective non-phosphorylated protein levels. Bars indicate the average of three independent experiments. Statistics (*t*-test): *P*<0.05. (**e**) Altering the levels of calcium-binding protein 39-like (CAB39L) and Sestrin-1 (SESN1) affected the response to everolimus. Graphs indicating the change in impedance of SUM159PT treated as indicated. (**f**) Metformin affected the clonogenic ability and potentiated the antitumorigenic effects of everolimus in both phosphatase and tensin homolog (PTEN) wt and PTEN K/D isogenic HCT116 cells. Briefly, HCT116 cells harboring either PTEN wt (left panel) or deleted for PTEN (right panel) were treated, 24 h after seeding, with metformin, in the presence or absence of everolimus. Histograms showing the average of three independent clonogenic assays. Colonies were stained after 9 days. Statistics (*t*-test): *P*<0.05. Please note that very similar results were obtained after altering the levels of the miR-21-5p, whose downregulation strictly mimicked the effect of metformin on the everolimus-treated cells (Supplementary Figure S5). (**g**) Invasion assay. HCT116 cells harboring either PTEN wt (left panel) or deleted for PTEN (right panel) were treated as from panel (**e**) for 24 h and the number of migrated cells was scored. Statistics (*t*-test): *P*<0.05.

Everolimus is a promising mTOR inhibitor shown to be effective in patients harboring hyperactive phosphoinositide-3 kinase pathway while not modifying the progression-free survival of patients harboring ‘normal’ phosphoinositide-3 kinase pathway activation in epidermal growth factor receptor 2-positive tumors[44]. As metformin treatment showed similar potency on both PTEN-proficient and -deficient cells, we assessed whether the anticancer activity of everolimus could be potentiated in PTENwt cells by the concomitant treatment with metformin. Regarding this hypothesis, we explored whether combining everolimus with metformin would exert synergistic effects. We found that metformin enhanced everolimus-induced killing of the PTENwt SUM159PT cells, as assessed by label-free viability assays (Figure 6a), combination index analysis of 72 h viability test (Figure 6b) and by clonogenic assay (Figure 6c). A similar effect was observed when the everolimus treatment was coupled with miR-21-5p antagonists; conversely, co-treatment with the miR-21-5p mimic counteracted the everolimus-induced cell killing of SUMPT159 cells (Supplementary Figure S5a and b). This was evident when comparing the 50% cytotoxic concentration (CC_50_) of the double-treated cells (Table 2). Biochemically, concomitant treatment with metformin and everolimus exhibited additive effects on the mTOR downstream effectors, especially with regard to inhibiting the phosphorylation of 4-EBP1 at thr^37/46^ and of S6 at Ser^235/236^ (Figure 6d). Again, treatment of the SUM159PT cells with the miR-21-5p agonist effectively counteracted those biochemical effects (Supplementary Figure S5c), in line with the previous observations. As CAB39L and SESN1 are the targets of metformin, we tested whether manipulating their levels could influence the response to everolimus. RNAi-mediated depletion of either CAB39L or SESN1 rendered SUM159PT cells more resistant to everolimus treatment, while ectopic expression of either CAB39L or SESN1 (this latter condition mimicking metformin treatment) increased cell death in response to the drug, with significant changes in the CC_50_ of the treated cells (Figure 6e and Table 2). Next we evaluated the effect of the combination treatment (metformin plus everolimus) on the matched PTEN-proficient and -deficient HCT116 cells (Figure 6f and g). This showed that the co-administration of metformin potentiated the effect of everolimus, which was significantly less potent toward PTENwt HCT116 cells when used alone (Figure 6f). We also evaluated the migration of the matched HCT116 cells co-treated with everolimus and metformin (Figure 6g). Even in this case, we observed synergy between the two drugs; however, this was less evident in the PTENdel HCT116 cells (Figure 6g), with both compounds less capable of inhibiting the migration. Altogether these findings indicate that downregulation of miR-21-5p expression by metformin, followed by release of CAB39L and SESN1, contributed to attenuating the mTOR aberrant activity and rendering breast cancer cells more prone to cell death induced by a clinically relevant mTOR inhibitor. Thus concomitant metformin and everolimus treatment may be exploited therapeutically.

## Discussion

**Figure 7:**
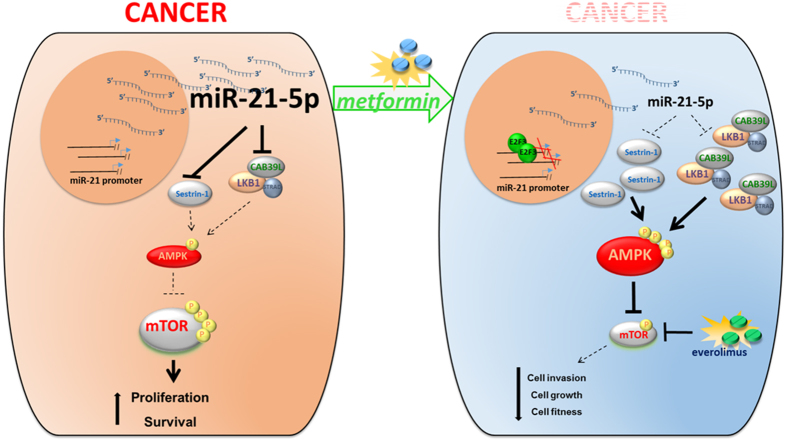
Working model. Metformin treatment downregulated, transcriptionally, the levels of miR-21-5p by inhibiting its transcription through increased occupancy of the promoter regions by E2F3. Consequently, this relieved the mir-21-5p-mediated repression of calcium-binding protein 39-like (CAB39L) and Sestrin-1 levels. This, in turn, activated AMP-activated protein kinase (AMPK) and inhibited mammalian target of rapamycin (mTOR) signaling, discouraging clonogenicity and invasion of the treated cells, independently of their phosphatase and tensin homolog (PTEN) status.

The biguanide metformin is the most widely used drug for treatment of type II diabetes. The primary systemic effect of metformin is the lowering of blood glucose levels achieved through reduced hepatic gluconeogenesis and increased glucose uptake in the skeletal muscles[45]. Metformin treatment leads to activation of AMPK *in vitro* and *in vivo* [46–48]. Epidemiological studies have demonstrated that chronical metformin treatment affects insurgence and mitigates the progression of several tumors. This has generated a plethora of reports addressing the potential anticancer mechanisms of the drug and the intracellular signaling it elicits, mainly downstream of AMPK activation; however, it should be noted that the growth-suppressive properties of metformin have ancient roots and that AMPK is not always required for the antiproliferative effects of metformin in cancer tissues[49]. In line with this, the anticancer effect of metformin does not appear only to be due to the control of glucose levels, as equally effective glucose-lowering-agents did not show relevant anticancer activity. Additionally, to date, the molecular mechanisms by which metformin leads to AMPK activation are not entirely understood. We and others have shown that metformin metabolic action resulted in a direct and broad modulation of several miRNAs that correlated with anticancer effects *in vitro* and *in vivo*. Starting from this observation, we focused here on the miR-21-5p, which we showed to be reduced by metformin in breast cancer cell lines, xenografted tumor tissues and in sera of metformin-treated breast cancer patients. We provided evidence that metformin modulates AMPK/mTOR signaling through miR-21-5p-dependent regulation of the scaffold proteins CAB39L and SESN1 (Figure 7). This happened, partially, through a metformin-dependent increase in the stoichiometry of the CAB39L–LKB1 complex. Interestingly, these effects were mimicked either by the overexpression of the targets (CAB39L and SESN1) or by downregulating miR-21-5p.

Then we further characterized, for the first time ever, the specific functional interaction between metformin, miR-21-5p and two effector targets, adding novelty to our understanding of the metabolic-anticancer properties of this promising compound. The involvement of CAB39L in mediating the activation of AMPK is already described[50]. With regard to SESN1, it is of note that this protein is a p53 target gene and is involved in linking DNA damage-induced p53 activation to the mTOR signaling pathway[11]. In this work, we showed upregulation of SESN1 in both p53wt (MCF-7) and p53mut (BT-474; BT-549) cells and in cells expressing undetectable p53wt (SUM159PT)[51]. This raises the interesting possibility that metformin-induced increase of SESN1 may be at least partially p53 independent and thus metformin treatment may be exploited in p53-null tumors. In line with this, Buzzai *et al.* [52] also described a selectivity of metformin treatment toward p53^−/−^ tumor cell growth. Of course, downregulation of the miR-21-5p cannot be the only anticancer mechanism of metformin. We have previously shown that upregulation of miR-33a and downregulation of c-MYC was relevant to metformin action in a similar experimental system[22]. In our experimental system, we did not observe changes in miR-33a levels upon downregulation of miR-21-5p, thus suggesting that the two mechanisms ignited by metformin may act independently. In line with this, we found that downregulation of miR-21-5p mainly took place at the transcriptional levels following increased occupancy of its promoter by E2F3. Conversely, upregulation of miR-33a followed increased levels of Dicer in metformin-treated cells. This might reflect a tumor- and stage-specific issue. We investigated whether metformin may synergize with everolimus, a clinical trial-grade mTOR inhibitor. This confirmed to be true in both breast cancer cell lines and in the isogenic colon carcinoma cells. Ectopic expression of CAB39L and SESN1 in everolimus-treated cells recapitulated the effect of metformin treatment, thus strengthening the relevance of the two miR-21-5p targets for the synergistic action of metformin. Altogether, this may represent a translationally relevant observation that may be worth testing in clinically relevant models. The latter will be a future avenue of investigation together with the possibility that the levels of miR-21-5p and those of CAB39L and SESN1 may inform selection of patients for combinatorial therapies.

## Materials and Methods

### Cell cultures and treatments

SUM159PT, MCF-7, BT-474 and BT-549 (ATCC, Manassas, VA, USA) were grown in Dulbecco’s modified Eagle’s medium (GIBCO, Thermo Fisher Scienific, Waltham, MA, USA) supplemented with 10% non-heat-inactivated fetal bovine serum (FBS; GIBCO) and 100 Units ml^−1^ Penicillin and 100 μg ml^−1^ Streptomycin (GIBCO). HCT116 PTEN wt and PTEN k/o cell lines (Horizon Discovery, Cambridge, UK) were grown in RPMI supplemented with 5% inactivated FBS (GIBCO) and 100 Units ml^−1^ Penicillin and 100 μg ml^−1^ Streptomycin (GIBCO). Metformin (1,1-dimethylbiguanide-hydrochloride) and everolimus (RAD001) were purchased from Sigma-Aldrich (St Louis, MO, USA) and SelleckChem (Houston, TX, USA), respectively.

### Chromatin immunoprecipitation

Precleared chromatin was incubated overnight with the following antibodies: anti-E2F3 (Santa Cruz Biotechnology #251, Dallas, TX, USA) and anti-Histone H4 (Upstate #06–866, EMD Millipore, Billerica, MA, USA). All the chromatin immunoprecipitation antibodies were used at 4 μg ml^−1^ of diluted chromatin. qPCR was used to assess for DNA enrichment. Primer sequences used for the three different E2F-binding sites contained in hsa-miR-21-5p promoter are: first site Fw: 5′-GCCCCAAGCTACCGTTTTTAA-3′, Rv: 5′-CCGGTTTGGCAACTGTAGCTA-3′; second site Fw: 5′-GCCTTTTGAGTCCAGGTGGTAA-3′, Rv: 5′-AGAAAAAGACACATTTGGGAAGAAA-3′; and third site Fw: 5′-GACTTACTGAGGTGACTTGAATATCTCC-3′, Rv: 5′-TTCAGCTATGGTAAGAGCCTTGG-3′.

### RNA processing and qPCR

Total RNA from breast cancer cell lines or tumor engrafment was extracted by TRI Reagent lysis reagent (Ambion, Thermo Fisher Scientific, Waltham, MA, USA), according to the manufacturer’s instructions. qPCR quantification of miRNA expression was performed using TaqMan MicroRNA Assays (Applied Biosystems, Thermo Fisher Scientific, Waltham, MA, USA) according to the manufacturer’s protocol. The first-strand cDNA was synthesized according to the manufacturer’s instructions (M-MLV RT Kit, Invitrogen, Thermo Fisher Scientific, Waltham, MA, USA). qPCR assays were performed by STEPOne PCR using the FastStart SYBR Green Master Mix (Applied Biosystems). Sequences of qPCR primers are ACTIN Fw: 5′-GGCATGGGTCAGAAGGATT-3′, Rv: 5′-CACACGCAGCTCATTGTAGAAGC-3′; pri-miR-21 Fw: 5′-TTTTGTTTTGCTTGGGAGGA-3′, Rv: 5′-AGCAGACAGTCAGGCAGGAT-3′; pre-miR-21 Fw: 5′-CATTGTGGGTTTTGAAAAGGTTA-3′, Rv: 5′-CCACGACTAGAGGCTGACTTAGA-3′; CAB39L Fw: 5′-TTAGTGCTCATCCTCATATCCTGTTTA-3′, Rv: 5′-CCCACAACGTAAGGCAATCTG-3′; and SESN1 Fw: 5′-ACTACATTGGAATAATGGCTGCGG-3′, Rv: 5′-CCCCAACCAACATGAGGAAATCAT-3′. RNU49 was used as an endogenous control to standardize miRNA expression. Taqman probes for the following miRNA was purchased: hsa-miR-21-5p (Applied Biosystems).

### Human serum specimen

Sera stored at −80 °C (*n*=96) were previously collected from de-identified patients with breast cancer enrolled in a randomized controlled trial to test the effect of different doses of metformin[38].

### Serum RNA extraction and qPCR

Total RNA from serum specimen was extracted by the miRNeasy Serum/Plasma Kit (Qiagen, Valencia, CA, USA), according to the manufacturer’s instructions. The first-strand cDNA was synthesized according to the manufacturer’s instructions (miScript Reverse Transcription Kit, Qiagen). qPCR assays were performed by STEPOne PCR (Agilent Technologies, Santa Clara, CA, USA) using the miScript SYBR Green PCR Kit (Qiagen). miScript primer Assay probe for the following miRNA was used: hsa-miR-21-5p. The *C. Elegans* miR-39 (miRNeasy Serum/Plasma Spike-In Control, Qiagen) was used as control to standardize the miRNA expression.

### Clonogenic assays

Cells were grown at 70% confluence and were exposed or not to a short pulse of metformin (0.5 mM). Subsequently, cells were detached and seeded at 600 cells per six-well into six-well dishes (Corning-Costar, New York, NY, USA) in drug-free media. Fresh media (25%) was added every 3 days. Number of colonies was scored by Cristal Violet staining 10 days later.

### Transwell invasion assay

Migration assay was performed using a 24-well Boyden chamber with a non-coated 8-mm pore size filter in the insert chamber (BD Falcon, Corning-Costar, New York, NY, USA). Cells were suspended in 0.5 ml Dulbecco’s modified Eagle’s medium/F12 media without containing FBS and seeded into the insert chamber. Cells were allowed to migrate for 24 h into the bottom chamber containing 0.5 ml of Dulbecco’s modified Eagle’s medium/F12 media containing 10% FBS in a humidified incubator at 37 °C in 5% CO_2_. Migrated cells were visualized by staining with 4,6-diamidino-2-phenylindole and counted. The average number of cells per field was expressed as the percentage of the control after normalizing for cell number.

### Wound-healing migration assay

SUM159PT cells were grown to 80% of confluence in six-well tissue culture plates and wounded with a sterile 10-μl pipette tip to remove cells. Phosphate-buffered saline washing was used to remove loosely attached cells. The progression of migration was photographed at different times under a light microscope. The number of cells migrated into the scratched area was calculated.

### Western blotting analysis

Cell lysis was performed on ice for 30 min in NP40 lysis buffer (50 mM Tris-HCl pH 7.4, 150 mM NaCl, 1% NP-40, 1 mM EGTA, 1 mM EDTA) supplemented with protease and phosphatase inhibitors (5 mM PMSF, 3 mM NaF, 1 mM DTT, 1 mM NaVO_4_). Equal amounts of total proteins extracts (30 μg) were resolved by 8% denaturing sodium dodecyl sulfate polyacrylamide gel electrophoresis and transferred for 2 h to polyvinylidene difluoride membrane. Membranes were blocked in 5% milk-TBS-0.05% Tween 20 for 1 h and incubated overnight with the specific primary antibodies. In the coimmunoprecipitation, the lysis buffer was modified accordingly the protein isoelectric point. Protein concentrations were determined by colorimetric assay (Bio-Rad, Hercules, CA, USA). For each immunoprecipitation, 1 μg of rabbit LKB1 (Abcam, Cambridge, UK, ab58786) or mouse CAB39L antibody (Abcam, ab57880) and 1 μg of rabbit or mouse IgG (Santa Cruz Biotechnology) as control were used. Precleared extracts were incubated with protein A/G-Agarose beads (Thermo Fisher Scientific, Rockford, IL, USA) in lysis buffer containing 0.05% bovine serum albumin and antibodies under constant shaking at 4 °C for 3 h. After incubation, agarose bead-bound immunocomplexes were rinsed with lysis buffer and eluted in 50 ml of sodium dodecyl sulfate sample buffer for western blotting. The following primary antibodies were used: anti phospho-AMPKα (Thr-172) (Cell Signaling, (Danvers, MA, USA) #2531); anti-AMPK (Cell Signaling, #9158); anti-phospho-mTOR (Ser-2448) (Cell Signaling, #2971); anti-β-Actin (Santa Cruz, sc-81178); anti CAB39L (Abcam, ab57880); anti SESN1 (Abcam, ab67156); anti LKB1 (Abcam, ab58786); anti IgG (Santa Cruz, sc-2030); anti-phospho p70 S6 Kinase (thr389) (Cell Signaling, #9234); anti-phospho 4-EBP1 (thr37/46) (Cell Signaling, #2855); and anti-phospho S6 (ser235/236) (Cell Signaling, #2211). All the indicated antibodies were used at the minimum dilutions suggested by the manufacturer. Secondary horseradish peroxidase-conjugated was purchased from Santa Cruz. ECL reagent (Amersham, GE Healthcare, Piscataway, NJ, USA) was employed for the chemo-luminescence detection. The Uvitec Alliance software (Eppendorf, Hamburg, Germany) was used to quantify the obtained data.

### Immunoprecipitation and LKB1 kinase activity

HEK-293 cells transfected with an LKB1 expression vector were treated or not with metformin for 24 h. Cells were lysed at 4 °C in Buffer A (Tris HCl pH 8 50 mM, EGTA 1 mM, EDTA 1 mM, NaF 50 mM, glicerolo 10%, KCl 5 mM, NaCl 170 mM, DTT 1 mM, protease inhibitor) plus 1% Triton X100. An equal volume of Buffer B (Tris HCl pH 8 50 mM, NaF 50 mM, glicerolo 7%, KCl 5 mM, MgCl_2_ 0.2 mM, CaCl_2_ 0.2 mM, protease inhibitor) was added after 20 min. Three milligrams of precleared cell lysate protein was incubated at 4 °C overnight with human LKB1 antibody.

The immune-precipitates were washed four times with Buffer B plus 0.15% NP40 and NaCl 100 mM. LKB1 activity was measured in a total volume of 50 μl consisting of 50 mM Tris HCl pH 7.5, 0.1 mM EGTA, 0.5 mM DTT, 10 mM MgCl2, 0.1 mM ATP and 200 uM of LKB1 substrate peptide[53] (Jena Bioscience, Jena, Germany, #PE-215). LKB1 phosphotransferase activity was assessed by using the ADP-Glo Kinase Assay (Promega, Madison, WI, USA, #V6930), according to the manufacturer’s instructions.

### EnSpire cellular label-free platform

SUM159PT, BT-549 and MCF-7 cells were seeded in specially designed 384-well plate with highly precise optical sensors able to measure changes in light refraction resulting from dynamic mass redistribution within the cell’s monolayer. Change in the light refraction was indicated by a shift in wavelength.

### Luciferase assays

For the 3′-UTR reporter assays, cells were co-transfected in 24-well plates PSICHECK2-CAB39L/or SESN1-3′UTR or PSICHECK2 (as a control) together with different doses of hsa-miR-21 mimic or control (Ambion, Thermo Scientific, Waltham, MA, USA). For the miR-21-5p promoter activity, the pMLuc-1 AccepTor plasmid (Novagen, Madison, WI, USA) was used for the generation of miR-21-5p promoter reporter constructs[21]. Renilla luciferase activities were measured 48 h after transfection using the Dual Luciferase Reporter Assay System (Promega) in the GloMax 96 Microplate Luminometer (Promega). Firefly luciferase was used to normalize the Renilla luciferase.

### Plasmid and transfection

The full-length 3′-UTR of the human CAB39L and SESN1 genes (OriGene, Rockville, MD, USA) were amplified and inserted downstream of the Renilla luciferase gene into the Not I site of the dual luciferase reporter plasmid psiCHECK-2 (Promega). CAB39L, SESN1 and miR-21-promoter mutants were made with the QuikChange site-directed mutagenesis Kit (Stratagene, San Diego, CA, USA) using the following primers: CAB39L Fw: 5′-GTGATGGGCAATTATAGAATGACGATTTATTAGAAAAAAATTGCTTTTATCAACATCTGATACAATGGCT-3′, Rv: 5′-AGCCATTGTATCAGATGTTGATAAAAGCAATTTTTTTCTAATAAATCGTCATTCTATAATTGCCCATCAC-3′; SESN1 Fw: 5′-GTAGTCGTAAATTTTTTTCGTTAAATCTAATCGGTTTTAATTCACGGATTTTCGTTAAATCTAA-3′, Rv: 5′-TTTTCGTTAAATCTAATTTTTTCGTACGTTTAAAAAATCTAATTTTTTCGTACGTTTACGGCTTT-3′; and miR-21-promoter Fw: 5′-CTTCTCCCCTCTGGGAAGTGAAATTACATTCACAGC-3′, Rv: 5′-GCTGTGAATGTAATTTCACTTCCCAGAGGGGAGAAG-3′. Transfections were performed with Lipofectamine 2000 or Lipofectamine RNAiMax (Life Technologies) according to the manufacturer’s recommendations, using either negative control (NC) (Life Technologies) or mimic-21-5p (Life Technologies) or miR-21-5p inhibitor (Life Technologies). SESN1 and E2F3 were silenced in SUM159PT cell line using siRNAs: siSESN1 5′-GGCATTAGAATTCCTCGA(dTdT)-3′ (Eurofins MWG, Louisville, KY, USA) and siE2F3 (1), 5′-CGUCCAAUGGAUGGGCUGC-3′[54] (Eurofins MWG), siE2F3 (2) 5′-UAACCUUUGAUUCUCUGAAUCCUCG-3′[55] (Eurofins MWG), respectively. siScr 5′-AAUUCAGCGUGUCCGGGGAG(dTdT)-3′ (Eurofins MWG) was used as a control while CAB39L was stably silenced in SUM159PT using shRNA expression vector (OriGene, TR314249). SUM159PT differently expressing high levels of CAB39L or SESN1 were obtained with a specific plasmid containing either CAB39L (OriGene, SC320905) or SESN1 (OriGene, SC212558) coding sequence. HEK293 cells expressing different levels of E2F3 were obtained by using a pCMV-Neo-Bam E2F3a[56]. It was a gift from Jacqueline Lees (Addgene plasmid # 37970, Cambridge, MA, USA).

### RNA-Seq

Total RNA from metformin- or vehicle-treated SUM159PT cells was isolated using miRNeasy including the optional DNase treatment (Qiagen). RNA quality was verified using an Agilent Bioanalyzer (Agilent Technologies; RNA 6000 Nano Kit). All RNAs used for subsequent library preparation had an RNA integrity number >9.0. RNA libraries for sequencing were generated according to the Epicenter ScriptSeq v2 RNA-Seq Library Preparation Kit (Illumina, Madison, WI, USA) with an initial ribosomal depletion step using Ribo-Zero Magnetic Gold (Illumina, Madison, WI, USA). Starting material was 5 μg of total RNA. Based on qPCR quantification, libraries were normalized to 1 nM and denatured by using 0.1 N NaOH. Cluster amplification of denatured templates was carried out according to the manufacturer’s protocol (Illumina, Inc., San Diego, CA, USA). Sequencing was performed on a Genome Analyzer IIx (Illumina) in paired-end mode; quality control has been performed to assess sequence data quality and to discard low quality reads. For the analysis, we exploited the RNA-Seq analysis workflow RAP that comprises of read mapping, transcript assembly and abundancy estimation followed by transcript-based differential expression via the Tuxedo suite. Paired-end reads were mapped to the human genome assembly hg19 with TopHat and further analyzed by the Cufflinks-Cuffdiff pipeline to identify differentially expressed transcripts. We run the pipeline without novel transcript discovery, correcting for multiply mapping reads with the ‘-u’ option of Cuffdiff and a mask file for rRNAs and tRNAs. The RNA-Seq was conducted with three biological replicates.

### Cell viability assay

Viability of treated cells was assessed using ATPlite assay (Perkin Elmer, Waltham, MA, USA) accordingly to the manufacturer’s instructions. Cells (8×10^2^ cells) were seeded in 96-well plates and cultured for 24 h and treated for 72 h with everolimus. Each plate was evaluated immediately on a microplate reader (Expire Technology, Perkin Elmer, Waltham, MA, USA).

### In silico individuation of miR-21-5p putative targets

Putative miR-21-5p targets were identified using the predictive target search of miRWalk with the programs DIANAmT, miRanda, miRDB, miRWalk, RNA22 and Targetscan, taking only those genes in consideration that were predicted to be miR-21-5p targets by at least five different programs and resulted as common deregulated in three different data sets. A permutation test and a false discovery rate procedure were used to assess a significant difference between tumoral and normal samples. Targets were sorted by a combined correlation *P*-value calculated with Stouffer’s and Fisher’s methods.

### Statistical analysis

For miRNA analysis, Pearson’s correlation coefficient was calculated to assess quality of replicates. Analysis of variance test (*α*=5%) was performed to assess statistical significance of the observed differences in miRNA profiling. Generally, Student’s *t*-test was used to assess significance of the data. *P*-values<0.05 were considered statistically significant. CompuSyn software (ComboSyn, Inc., Paramus, NJ, USA) was used for CC_50_ determination.

### Immunohistochemical analysis

Formalin-fixed and paraffin-embedded 5 μm sections from mice tumor sections were stained with anti-CAB39L antibody (Abcam, ab57880). Seven fields chosen randomly from each sample were scored.

## Figures and Tables

**Table 1:** Commonly deregulated and predicted gene targets of miR-21-5p in three different patient data sets

*Gene*	*n softw pred.*	*Gastric mRNA*			*Head/neck miRNA/mRNA*					*TGCA breast miRNA/mRNA*					*Combined* P*-value*	*Combined* P*-value Stouffer*
		P*-value T/N*	*FDR*	*Log2fold T/N*	R *Pearson*	P*-value Pearson*	P*-value T/N*	*FDR*	*Log2fold T/N*	R *Pearson*	P*-value Pearson*	P*-value T/N*	*FDR*	*Log2fold T/N*		
***CAB39L***	**5**	**0.0001152**	**0.0019075**	**−1.4499698**	**−0.760**	**1.133E-06**	**1.188E-05**	**0.002008**	**−2.026**	**−0.226**	**7.82E-08**	**1.92E-05**	**2.31E-05**	**−0.8002**	**3.859E-35**	**0**
***SESN1***	**4**	**0.0051602**	**0.0172594**	**−0.9251614**	**−0.401**	**0.0280352**	**0.0020789**	**0.0449665**	**−0.877**	**−0.312**	**5.68E-14**	**0.048219**	**0.0151231**	**−1.1244375**	**4.821E-20**	**0**
*CDH19*	5	0.0025804	0.0110663	**−**0.9266954	**−**0.669	5.281E**-**05	0.0046097	0.0723101	**−**0.723	**−**0.187	8.99E-06	0.0044263	0.0019835	**−**1.7027083	2.646E**-**18	5.551E**-**17
*PLP1*	4	7.931E**-**05	0.0016367	**−**1.2054348	**−**0.729	4.952E**-**06	4.833E**-**06	0.0014273	**−**1.150	**−**0.284	9.99E-12	0.0451993	0.0143104	**−**1.504125	3.314E**-**17	5.551E**-**17
*SCP2*	4	0.001315	0.0073171	**−**0.7303324	**−**0.427	0.018561	0.0009213	0.0265006	**−**0.573	**−**0.311	6.58E-14	0.0046633	0.0020689	**−**0.6649583	4.757E**-**16	5.211E**-**13
*GREM2*	5	6.857E**-**06	0.0010567	**−**2.0507565	**−**0.399	0.0288326	3.02E**-**05	0.0033958	**−**1.263	**−**0.178	2.59E-05	0.0325748	0.0108243	**−**1.7469698	2.162E**-**14	1.155E**-**11
*CNTFR*	6	0.0064167	0.019916	**−**0.6214109	**−**0.492	0.0057344	0.0053505	0.0785863	**−**0.418	**−**0.366	5.04E-19	2.96E-07	1.94E-06	**−**0.656	2.474E**-**13	8.092E**-**12
*SLC16A7*	3	1.304E**-**06	0.0009245	**−**3.042192	**−**0.592	0.0005743	4.05E**-**06	0.0012815	**−**1.316	**−**0.415	1.43E-24	0.030846	0.0103129	**−**2.09625	4.504E**-**13	1.82E**-**12

Abbreviations: comb., combination; FDR, false discovery rate; N, normal; n.softw.pred., number of prediction software; T, tumor.

A permutation test and a FDR procedure were used to assess a significant difference between tumoral and normal samples. A Pearson’s correlation coefficient for the miR-21-5p/mRNA pairs in matched samples of gastric, head and neck and breast cancers was evaluated. Targets were sorted by a combined correlation *P*-value calculated with Stouffer’s and Fisher’s methods. In bold are reported the predictive genes with the lowest p-value.

**Table 2:** CC_50_ values

	*CC*_*50*_ *(nM)*
Vehicle	16.47±0.5
Metformin	7.88113±0.36
21 inhibitor	6.93223±0.49
Mimic 21	42.2588±0.47
Si SESN1	58.6414±0.36
Over SESN1	12.7958±0.38
Sh CAB39L	54.8008±0.3
Over CAB39L	12.7175±0.42
Sh CAB39L si SESN 1	81.3338±0.45
Sh CAB39L over SESN 1	11.0902±0.4
Sh CAB39L over CAB39L	8.68656±0.3
OverCAB39L over SESN1	3.30453±0.29

Abbreviations: CAB39L, calcium-binding protein 39-like; CC_50_, 50% cytotoxic concentration; SESN1, Sestrin-1. Altering the levels of CAB39L and/or SESN1 affected everolimus response. Here are reported the CC_50_ values obtained from viability assay experiments conducted in SUM159PT cells treated with everolimus (0.001–100 nM) for 72 h. CompuSyn software (http://www.combosyn.com/feature.html) was used for analysis.
