# Author Correction: Metformin-induced ablation of microRNA 21-5p releases Sestrin-1 and CAB39L antitumoral activities

**DOI:** 10.1038/s41421-024-00655-2

**Published:** 2024-03-13

**Authors:** Claudio Pulito, Federica Mori, Andrea Sacconi, Frauke Goeman, Maria Ferraiuolo, Patrizia Pasanisi, Carlo Campagnoli, Franco Berrino, Maurizio Fanciulli, Rebecca J. Ford, Massimo Levrero, Natalia Pediconi, Ludovica Ciuffreda, Michele Milella, Gregory R. Steinberg, Mario Cioce, Paola Muti, Sabrina Strano, Giovanni Blandino

**Affiliations:** 1 Molecular Chemoprevention Unit, Italian National Cancer Institute ‘Regina Elena’, Rome, Italy; 2 Oncogenomic and Epigenetic Unit, Italian National Cancer Institute ‘Regina Elena’, Rome, Italy; 3 Department of Preventive & Predictive Medicine, Fondazione IRCCS Istituto Nazionale Dei Tumori, Milan, Italy; 4 Unit of Endocrinological Gynecology, Ospedale Sant’Anna di Torino, Turin, Italy; 5 SAFU, ‘Regina Elena’ National Cancer Institute, Rome, Italy; 6 Division of Endocrinology and Metabolism, Department of Medicine, McMaster University, Hamilton, ON Canada; 7 Epigénétique et Épigénomique des Carcinomes Hépathocellulaires Viro-Induits du Centre de Recherche en Cancérologie de Lyon, Lyon, France; 8 Department of Molecular Medicine, Sapienza University of Rome, Rome, Italy; 9 Division of Medical Oncology A, Italian National Cancer Institute ‘Regina Elena’, Rome, Italy; 10 Department of Oncology, Juravinski Cancer Center, McMaster University, Hamilton, ON Canada

Correction to: *Cell Discovery* (2017) 3: 17022

10.1038/celldisc.2017.22; published online 4 July 2017

Relative to chapter: Modulation of CAB39L and SESN1 impinged on clonogenicity and invasiveness of breast cancer cells. We have evaluated the consequences of metformin-miR-21-5p modulation of CAB39L and SESN1 on the clonogenic activity of SUM159PT triple-negative breast cancer cell line (Fig. 4g and Supplementary Fig. S4e). During the generation of the representative images in Fig. 4g (and therefore Supplementary Fig. S4e which is the same experiment), the cell colony staining images from Fig. 2h, 0.5 and 1 nM miR-21-5p inhibitor treatments were inadvertently copied and pasted to represent the *metformin* + *shCAB39L(A)* and *metformin* + *siSESN1* treatment conditions of Fig. 4g, respectively. The incorrect images in Fig. 4g and Supplementary Fig. S4e have been replaced by the images from the correct treatment groups. This change does not impact the results because the data with their statistical significance described in the manuscript are based on the correct colony staining images for the assigned treatments.

**Figure:**
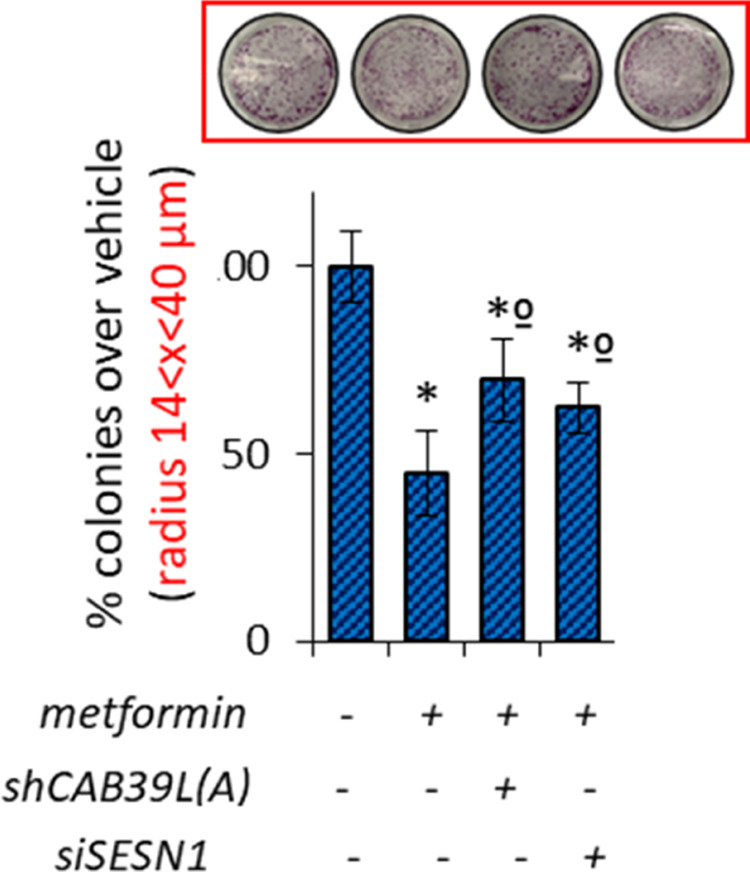
**Figure 4 g** Histograms showing average colony counts (radius 14 < *x* < 40 μm) of SUM159PT cells transfected either with a control vector or CAB39L- or SESN1-targeting shRNAs and treated with vehicle or metformin (0.5 mM). Histograms indicating the average ± s.e. of triplicate experiments. Statistics (*t*-test): *P* < 0.05.

**Figure:**
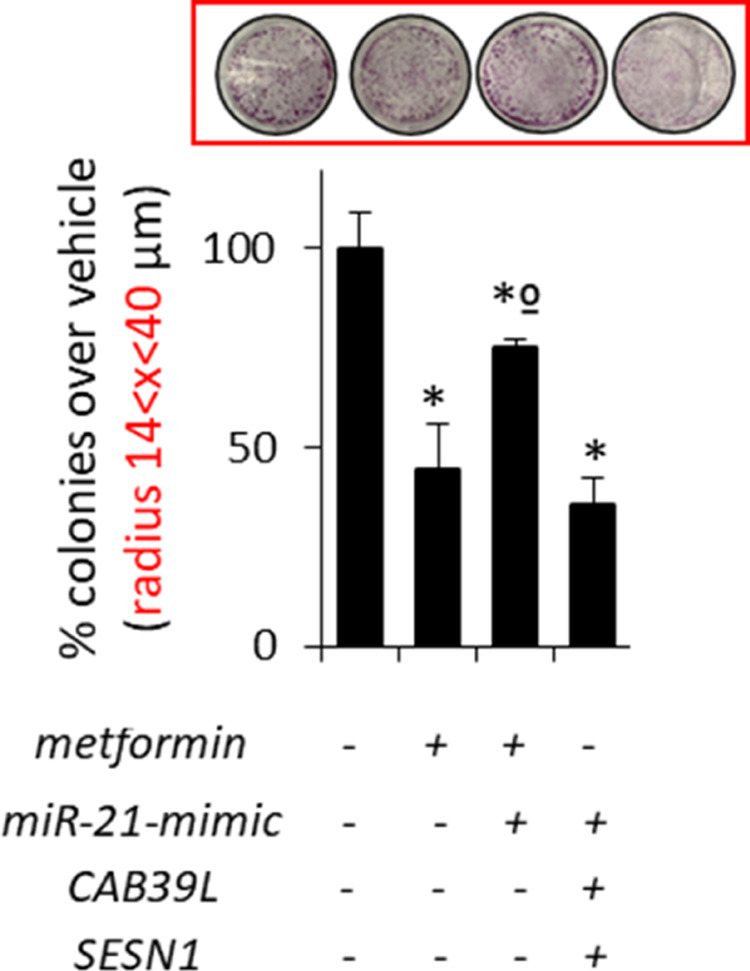
**Supplementary Figure S4 e** Histograms showing average colony counts (radius 14 < *x* < 40 μm) of SUM159PT cells transfected either with a control vector or CAB39L- or SESN1 over-expressing vector or mimic miR-21 vector and treated with vehicle or metformin (0.5 mM). Histograms indicating the average ± s.e. of triplicate experiments. Statistics (*t*-test): *P* < 0.05.

Please note that the histograms of Supplementary Fig. S4e belong to the experiments reported in Fig. 4g.

Relative to chapter: CAB39L and SESN1 are modulated by metformin-miR-21-5p. We have evaluated the ability of the inhibition of miR-21-5p to inhibit SUM159PT cell colony formation (Fig. 2h). Cell staining images included in Fig. 2h are correctly representative of the results reported in each column of the shown histogram.

Relative to chapter: Biochemical effects of metformin-induced increase of CAB39L and SESN1. In Fig. 3a and b, we have evaluated the effects of metformin treatment on the protein levels of CAB39L and SESN1 in SUM159PT cells and the relative activation of AMPK, mTOR signalling, S6 and 4-EBP1 (Fig. 3a and b). Fig. 3a left and right panels were derived from two independent western blotting analyses as indicated. We acknowledge that there are similarities between AMPKα lanes 3 and 4. We replaced the right panel of the AMPKα blot with the correct one. We also provided uncropped original gels of the indicated western blot panels. This change does not impact the results already published.

**Figure:**
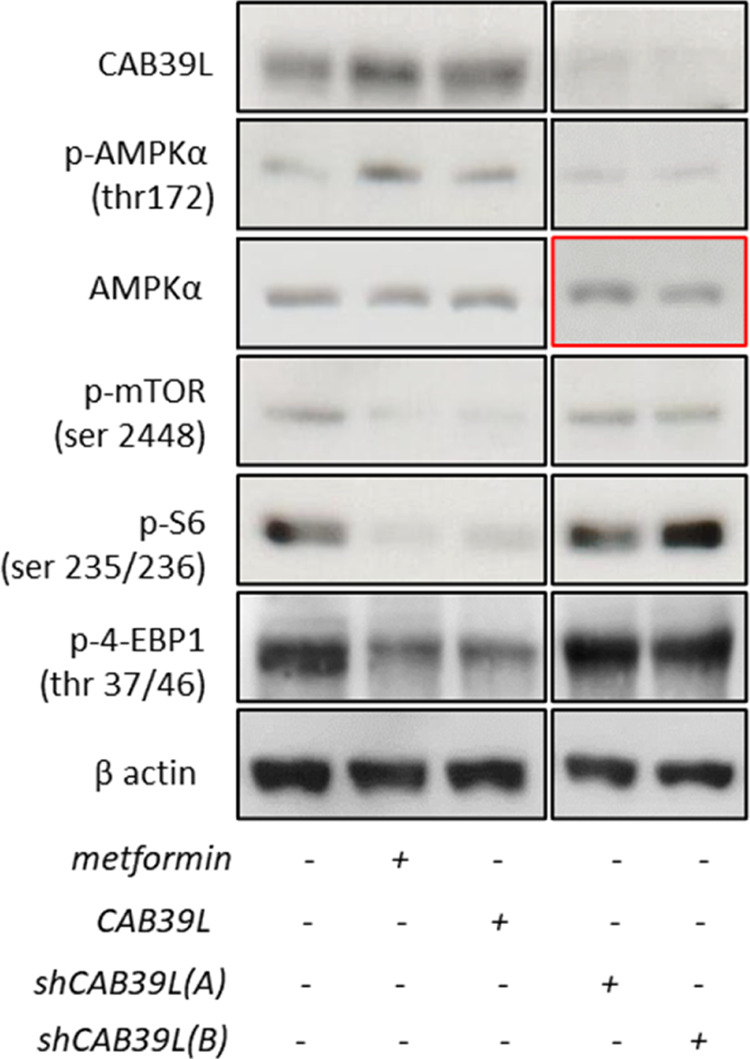
**Figure 3 a** Representative western blotting of whole-cell lysates from SUM159PT treated with vehicle or metformin or transfected with the expression vectors coding for CAB39L or for two shRNAs against CAB39L, stained with the indicated pan- and phospho-specific antibodies.

Long-exposure
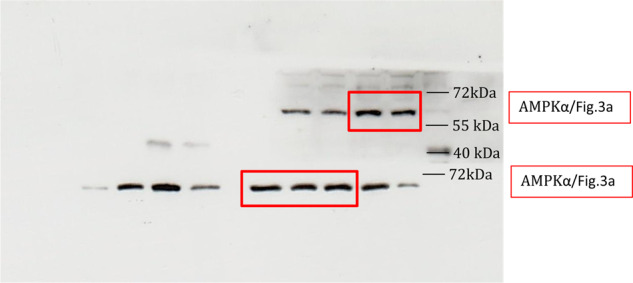


Short-exposure
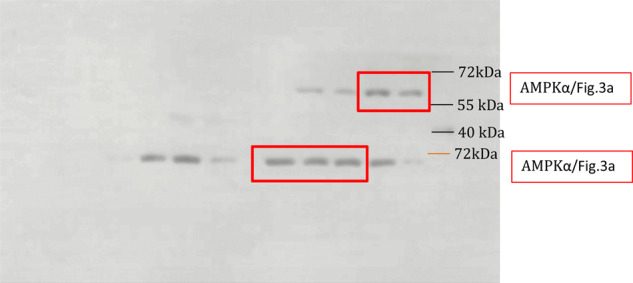


**Figure:**
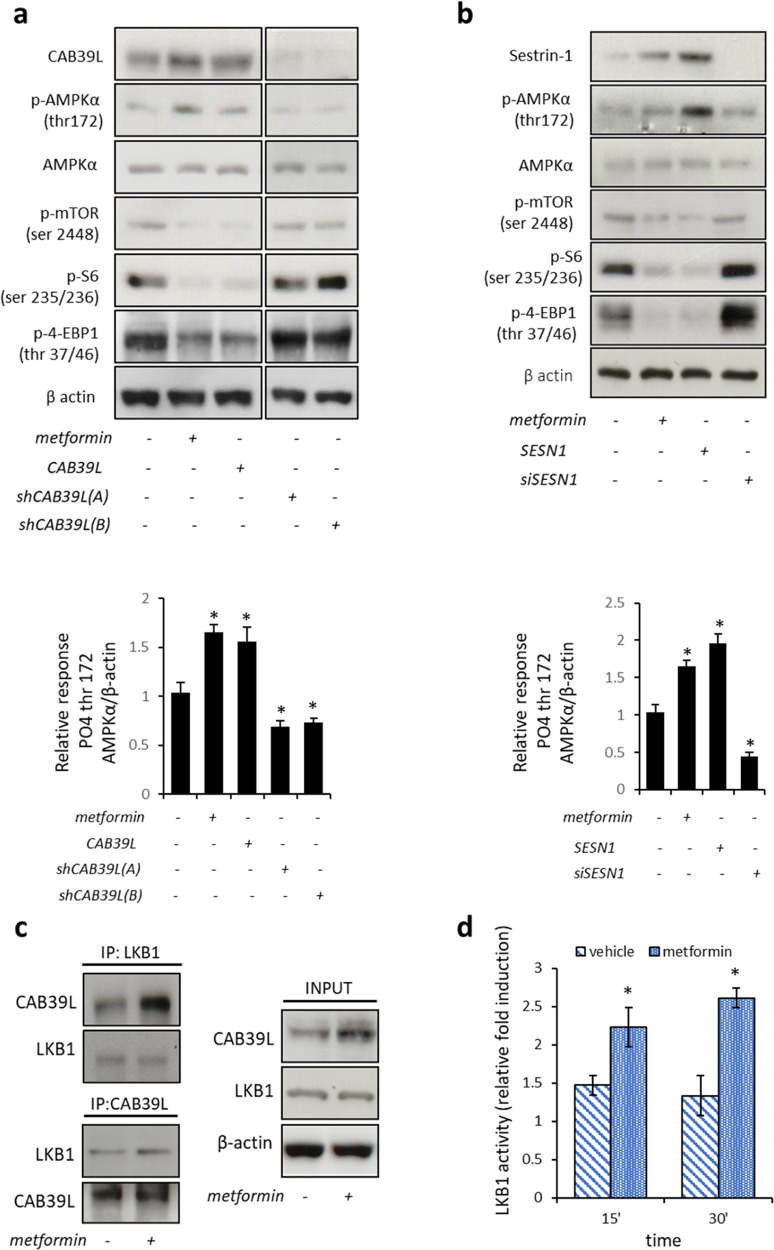
**Figure 3** Calcium-binding protein 39-like (CAB39L) and Sestrin-1 (SESN1) mediated the anticancer effects of metformin through AMP-activated protein kinase–mammalian target of rapamycin (AMPK-mTOR) signaling. (**a**, **b**) Altering the levels of CAB39L and SESN1 mimicked the effect of metformin on the AMPK/mTOR signaling axis. (**a**) Upper panel. Representative western blotting of whole-cell lysates from SUM159PT treated with vehicle or metformin or transfected with the expression vectors coding for CAB39L or for two shRNAs against CAB39L, stained with the indicated pan- and phospho-specific antibodies. Lower panel. Quantitative densitometry of phospho-thr^172^ AMPKα calculated from the analysis of three western blottings including that in panel (**a**). (**b**) Upper panel. Representative western blotting of similarly stained whole-cell lysates from of the same cells treated as from panel (**a**) and transfected with the expression vectors for SESN1 or with a siRNA against SESN1. Lower panel. Quantitative densitometry of phospho-thr^172^ AMPKα calculated from the analysis of three western blottings including that in panel (**b**). (**c**) The LKB1–CAB39L complex was modulated by metformin. Representative western blotting of whole-cell lysates of vehicle- and metformin-treated SUM159PT cells, immunoprecipitated with anti-LKB1 and anti-CAB39L antibodies (upper left and lower left panels, respectively). Actin staining was used as a loading control for the input material (right panel). (**d**) Metformin stimulates LKB1 kinase activity. Immuno-kinase assay. Histograms showing the enzymatic activity of LKB1: immunoprecipitates from HEK-293 cells treated or not with 0.5 mM of metformin for 24 h. Mean of two independent experiments. Statistics (*t*-test): *P* < 0.001.

**Figure:**
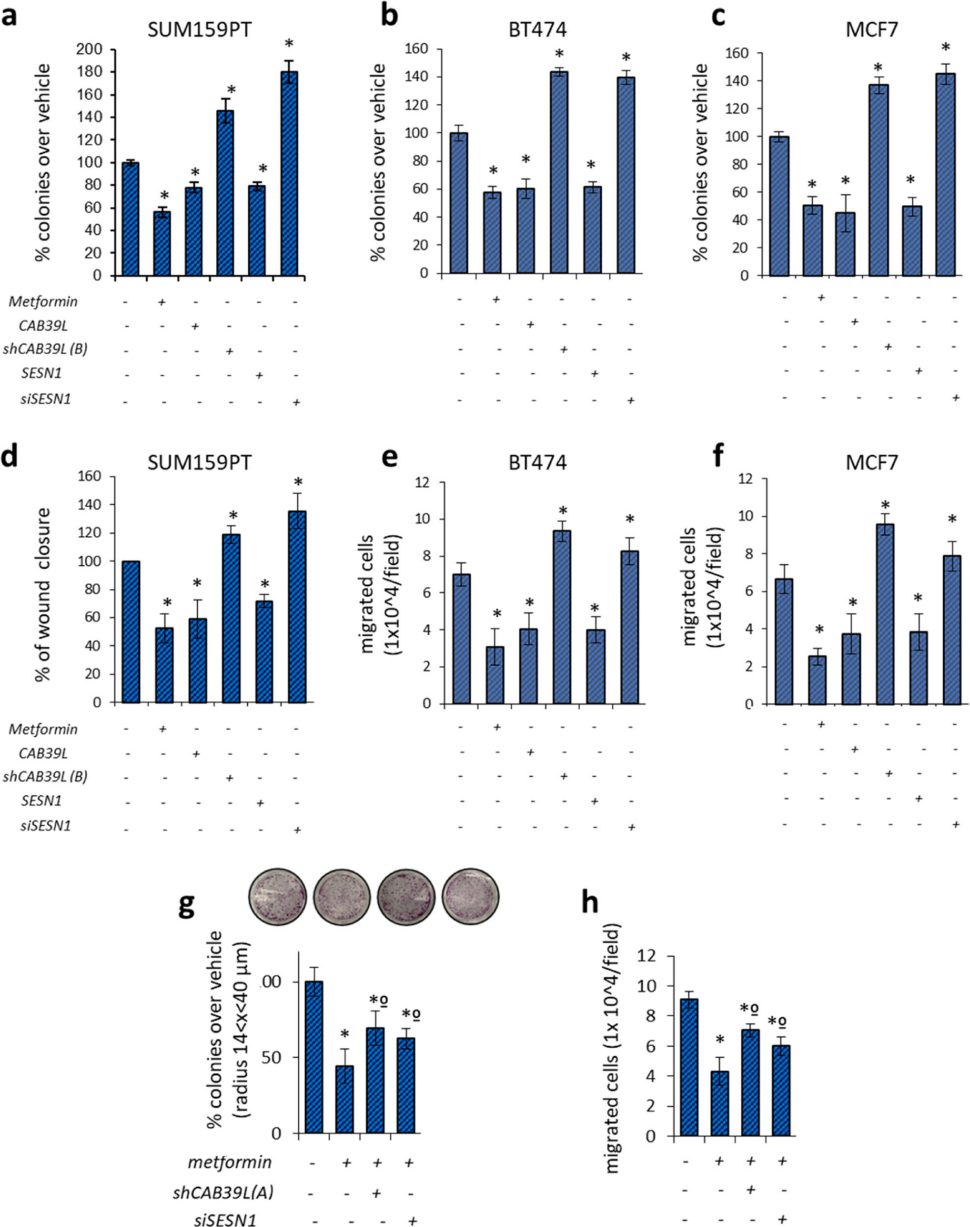
**Figure 4** Modulation of calcium-binding protein 39-like (CAB39L) and Sestrin-1 (SESN1) underlies metformin anticancer activities. (**a**–**c**) Colony-forming assay. Histograms showing average colony counts of SUM159PT (**a**), BT-474 (**b**) and MCF-7 (**c**) cells expressing either a control vector or a CAB39L-expressing vector or CAB39L- or SESN1-targeting shRNAs and treated with vehicle or metformin (0.5 mM) before seeding at clonal density. Statistics (*t*-test): *P* < 0.05. (**d**) Wound healing assay. Percentage of wound closure (over vehicle) of SUM159PT cells transfected and treated as from panel (**a**–**c**), for 48 h. Statistics (*t*-test): *P* < 0.05. (**e**, **f**) Invasion assay. BT-474 (**e**) and MCF-7 (**f**) cells were treated with vehicle or metformin (0.5 mM) for 24 h and the number of migrated cells was scored. (**g**, **h**) Colony-forming assay (**g**) and invasion assay (**h**). Histograms showing average colony counts (**g**) or the number of migrated cells (**h**) of SUM159PT cells transfected either with a control vector or CAB39L- or SESN1-targeting shRNAs and treated with vehicle or metformin (0.5 mM). Histograms indicating the average ± s.e. of triplicate experiments. Statistics (*t*-test): *P* < 0.05. *Significant versus vehicle, ºSignificant versus untransfected, metformin-treated ones.

**Figure:**
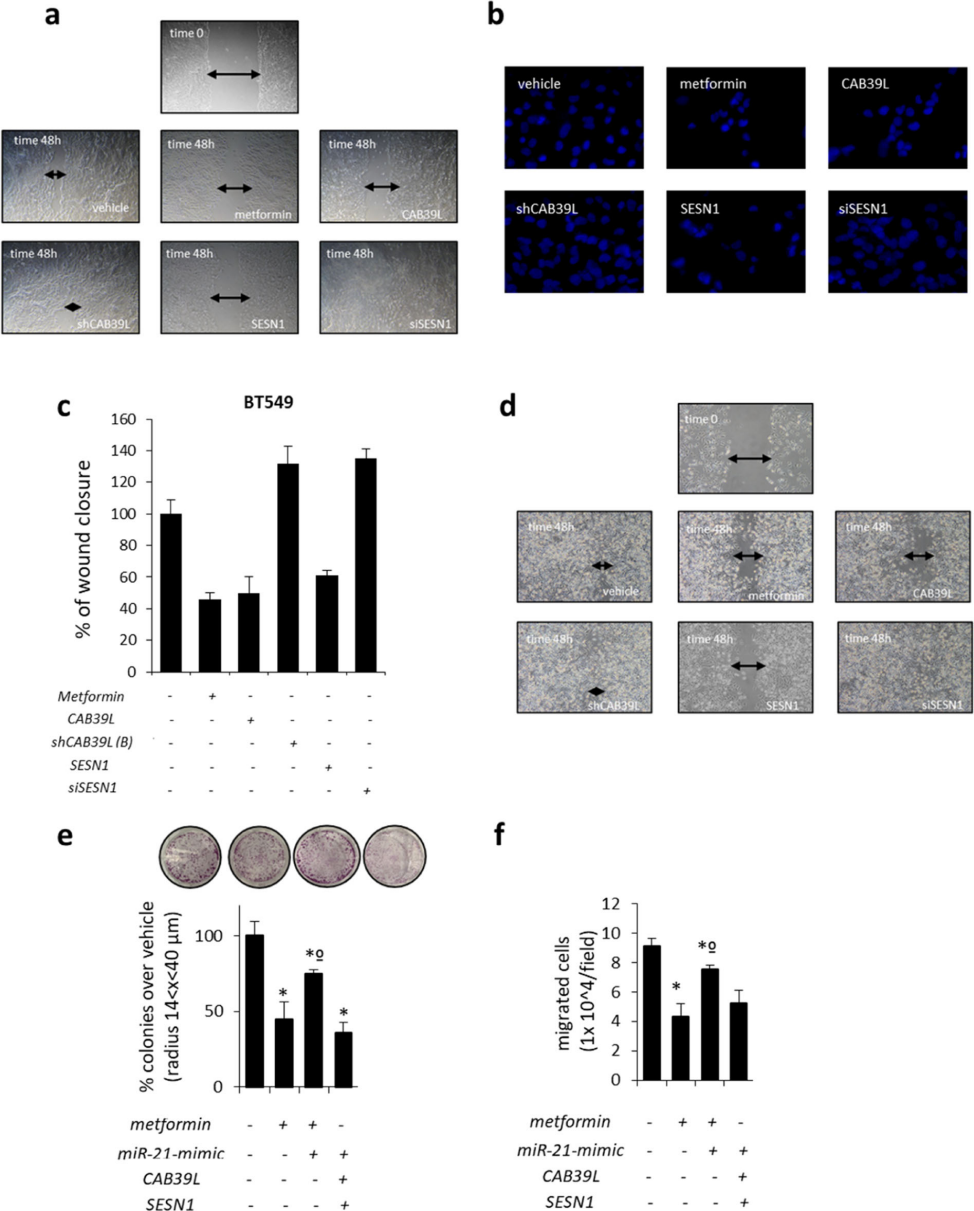
**Supplementary Figure S4** (**a**) Representative micrographs of wound healing closure of SUM159PT as reported in (**d**). (**b**) Representative micrographs of migrated BT-474 cells as reported in (**e**). (**c**) Wound healing assay. Percentage of wound closure (over vehicle) of BT-549 cells expressing either a control vector or a CAB39L expressing vector or CAB39L- or SESN1-targeting shRNAs and treated with vehicle or metformin (0.5mM). Statistics (*t*-test): *p* < 0.05. (**d**) Representative micrographs of wound healing closure of BT549 from supplementary (**c**). (**e**, **f**) Colony assay (**e**) and invasion assay (**f**). Histograms showing average colony counts (**e**) or the number of migrated cells (**f**) of SUM159PT cells treated with vehicle or metformin and/or transfected with CAB39L and SESN1 expressing vector and/or miR-21-5p agonist construct. *significant versus vehicle, ºsignificant versus untrasfected, metformin-treated ones.

