# Supplementary material for: Metformin-induced ablation of microRNA 21-5p releases Sestrin-1 and CAB39L antitumoral activities
Source: Cell Discov. 2017 Jul 4;3:17022–. doi: 10.1038/celldisc.2017.22 (PMC5501975; doi:10.1038/celldisc.2017.22)

Legends to Supplementary Figures

Supplementary Figure S1. (related to Figure 1) (**a**) Heatmap depicting the microRNAs differentially modulated in SUM159PT cells treated with vehicle or with metformin (0.5mM), for 24 h. The blue arrow indicates the miR-21-5p. (**b**) Quantitative-PCR: levels of miR-21-5p from BT-474, MCF-7 and BT-549 cells treated with metformin for 24 h. Bars indicate the average of three independent experiments. Statistics (t-test): $p<0.05$. (**c**) Altering the levels of E2F3 affects the metformin-mediated inhibition of miR-21-5p expression. Expression of miR-21-5p in SUM159PT cells silenced or not with two different E2F3 siRNAs and treated with metformin (0.5mM) for 24 hrs. Statistics (t-test): $p<0.05$. (**d-e**) E2F3 binding to the miR-21-5p promoter mediated the effect of metformin. Histograms showing the amplification of the E2F binding sites in the chromatin precipitated with anti-E2F3 and anti-acetylated Histone H4 (Acetyl-H4), from vehicle and metformin-treated (0.5mM, 24h) SUM159PT cells. Statistics (t-test): $p<0.05$. Ns: not significant. (**f-i**) Altering the levels of the miR-21-5p mimicked, oppositely, the effect of metformin treatment. Left panels. Histograms showing the average percentage of colonies formed by SUM159PT (**f**), BT-549 (**g**), BT-474 (**h**) and MCF-7 (**i**) cells expressing either a control vector or a miR-21-5p mimic vector or a miR-21-5p inhibitor vector and treated with vehicle or metformin (0.5mM) for 24 h. Statistics (t-test): $p<0.05$. Right panels. Histograms showing the number of migrated cells from the cells transfected as mentioned before and treated with vehicle or metformin (0.5mM) for 24 h. Statistics (t-test): $p<0.05$.

Supplementary Figure S2. (related to Figures 1 and 2) Modulation of CAB39L and SESN1 by metformin *in vitro* and *in vivo*. (**a**) RNAseq. Upper panel. Normalized expression values (FPKM) of CAB39L and SESN1 transcripts in SUM159PT cells treated for 24 h with vehicle or metformin (0.5mM). Lower panel. Genome Browser snapshot of PE-RNA-Seq reads of a representative sample showing the genomic regions of CAB39L and Sestrin-1 in absence/presence of metformin. (**b**) Left panel. Representative micrographs of the tumor sections from vehicle- or metformin- treated mice stained with CAB39L antibodies. Right panel. Histograms showing the percentage of CAB39L positive cells (10 fields/section averaged). Error bars represent mean ± SE. Statistics (t-test): $p<0.05$. (**c**) CAB39L and SESN1 levels are prognostically relevant for breast cancer patients. Kaplan-Meier curves indicating the probability of breast cancer recurrence in women expressing high or low levels of either CAB39L (left) or SESN1 (right) (http://kmplot.com/analysis/).

Supplementary Figure S3. (related to Figure 3). Altering the levels of either of miR-21-5p or CAB39L/SESN1 affects the effect of metformin treatment on AMPKα and mTOR activation. (**a**) Representative western blotting of whole cell lysates from HEK-293 cells treated with vehicle or metformin and/or transfected with CAB39L- or SESN1-targeting shRNAs/siRNA. (**b**) Quantitative densitometry of phospho-thr^{172} AMPKα and phosphor-ser^{2448} mTOR calculated from the analysis of three western blots including that in (a). (**c**) Representative western blotting of whole cell lysates from HEK-293 cells treated with vehicle or metformin and transfected with CAB39L and SESN1 expressing vectors. (**d**) Upper panel. Representative western blotting of whole cell lysates from HEK-293 cells treated with vehicle or metformin and/or transfected with CAB39L and SESN1 expressing vector and/or miR-21-5p agonist construct in absence of metformin. Lower panel. Quantitative densitometry of phospho-thr^{172} AMPKα and phosphor-ser^{2448}. (**e**) Colony forming assay. Histograms showing average colony counts of HEK-293 cells expressing either a control vector or CAB39L- or CAB39L or LKB1 targeting shRNAs/siRNA and treated with vehicle or metformin (0.5mM) before seeding at clonal density. Histograms indicating the average ± SE of duplicate experiments Statistics (t-test): $p<0.05$.

Supplementary Figure S4. (related to Figure 4). (**a**) Representative micrographs of wound healing closure of SUM159PT as reported in (d). (**b**) Representative micrographs of migrated BT-474 cells as reported in (e). (**c**) Wound healing assay. Percentage of wound closure (over vehicle) of BT-549 cells expressing either a control vector or a CAB39L expressing vector or CAB39L- or SESN1-targeting shRNAs and treated with vehicle or metformin (0.5mM). Statistics (t-test): $p<0.05$. (**d**) Representative micrographs of wound healing closure of BT549 from supplementary (c). (**e**, **f**) Colony assay (**e**) and invasion assay (**f**). Histograms showing average colony counts (**e**) or the number of migrated cells (**f**) of SUM159PT cells treated with vehicle or metformin and/or transfected with CAB39L and SESN1 expressing vector and/or miR-21-5p agonist construct. * significant versus vehicle, º significant versus untrasfected, metformin-treated ones.

Supplementary Figure S5. (related to Figure 6). Altering the miR-21-5p levels mimicked the effect of metformin in everolimus treated cells. (**a**) Graphs indicating the fitness (impedance) of SUM159PT, MCF-7 and BT-549 cells transfected with a miR-21-5p mimic vector, or a miR-21-5p inhibitor vector and subsequently treated with everolimus (10nM) for

0–550 minutes, as assessed by a label free assay. Statistics (t-test): $p<0.05$. (**b**) Histograms showing the average percentage of colonies of SUM159PT cells transfected with either a control or a miR-21-5p inhibitor expressing vector and subsequently treated with everolimus (0-100nM). Bars indicate the mean ± SE of three independent experiments. Statistics (t-test): $p<0.05$. (**c**) Representative western blotting (upper panel) and quantitative densitometry (lower panel) of the protein bands corresponding to (phospho-ser^{2448})-mTOR, (phospho-thr^{389})-p70 S6, (phospho-ser$^{235/236}$)-S6 and (phospho-thr$^{37/46}$)-4-EBP1, normalized to their respective non-phosphorylated protein levels. Bars indicate the mean +/- SE of three independent experiments. Statistics (t-test): $p<0.05$.

Supplementary Figure S1

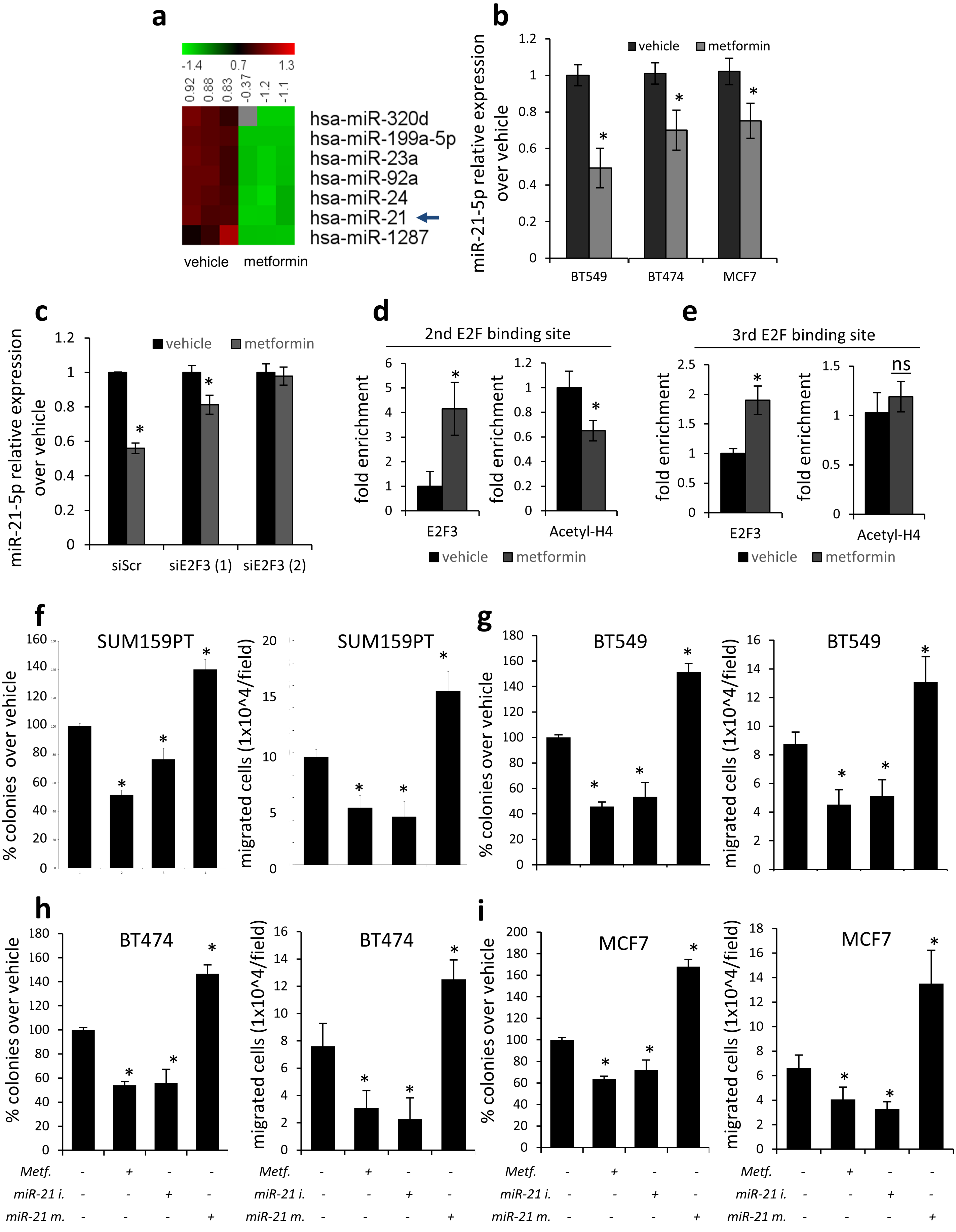

Supplementary Figure S2

a

Gene	Transcript	FPKM vehicle	FPKM Metformin	log2	pvalue	qvalue
CAB39L	NM_001079670	1.40	1.94	0.47	0.0016	0.0075
SESN1	NM_001199933	0.75	4.81	2.67	0.0001	0.0003

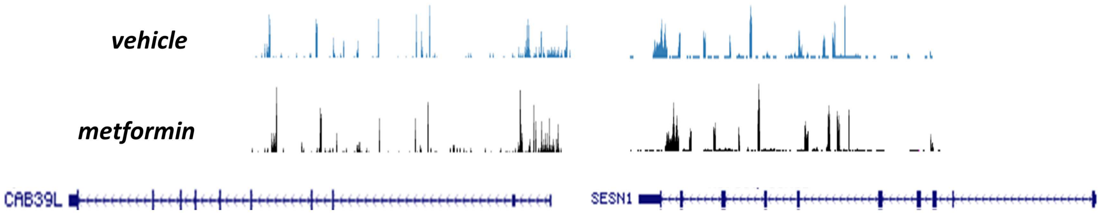

b

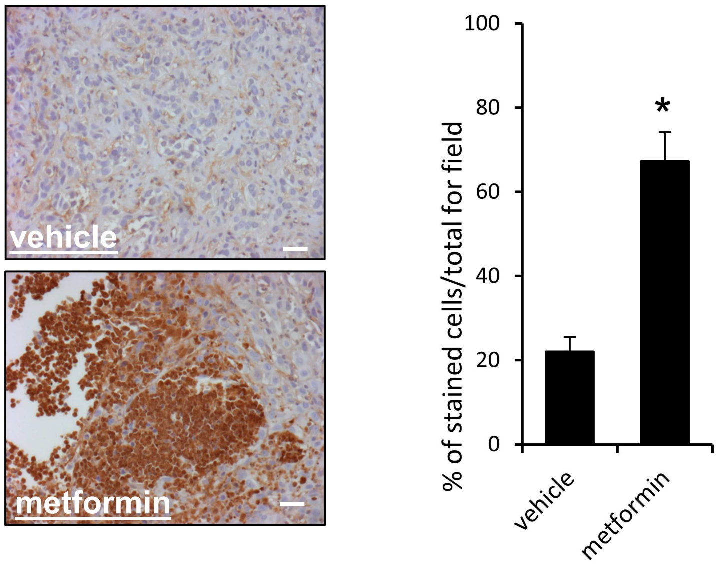

c

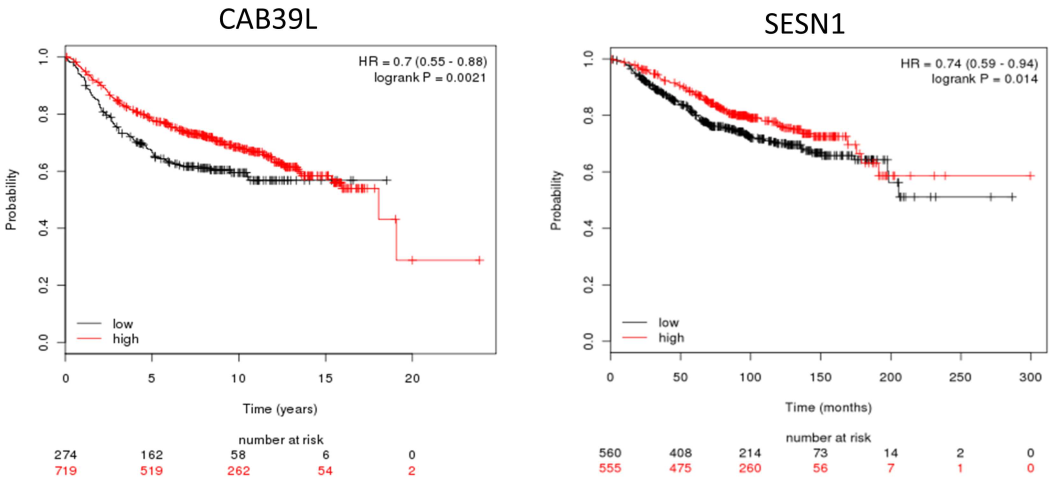

Supplementary Figure S3

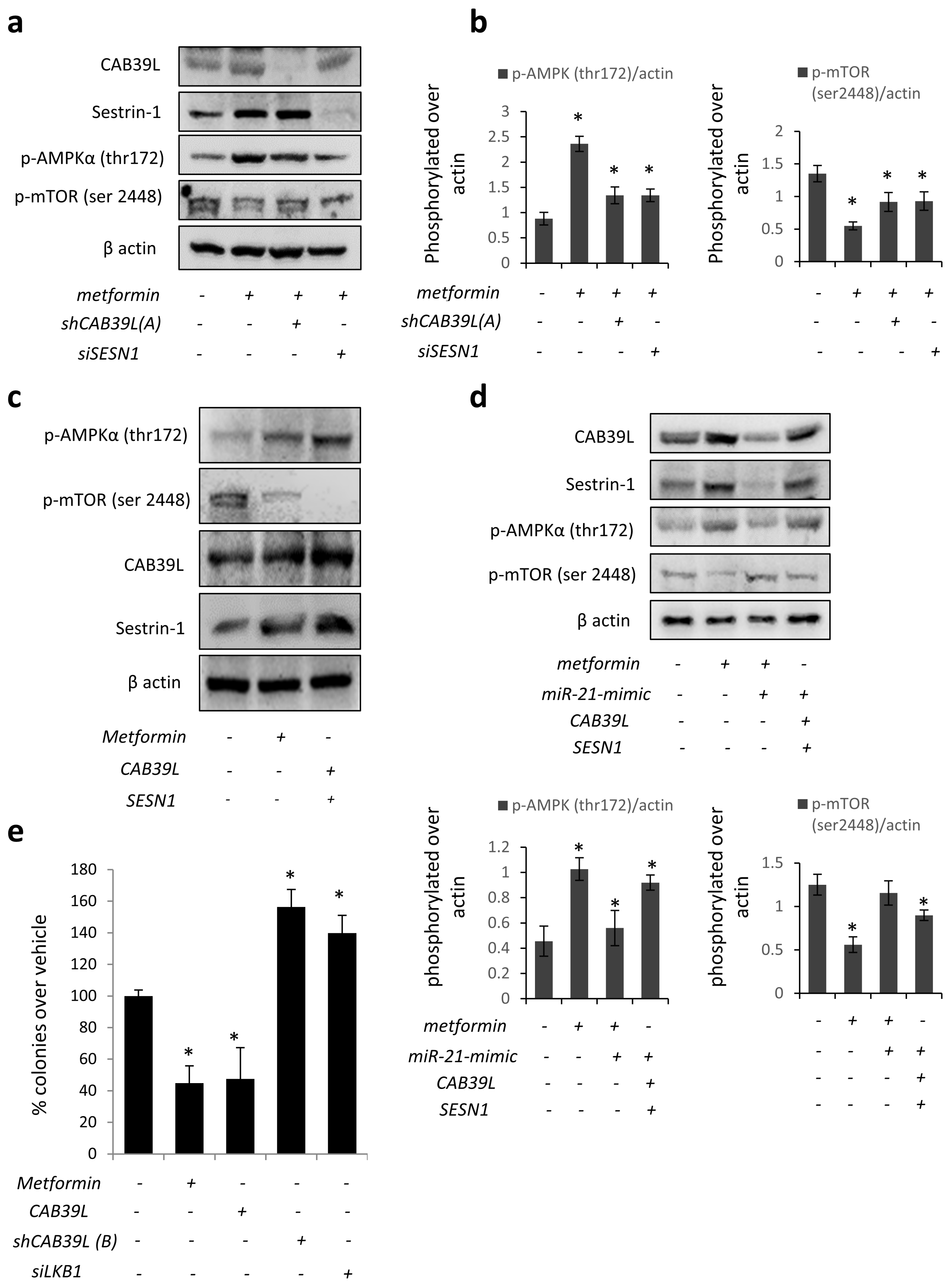

Supplementary Figure S4

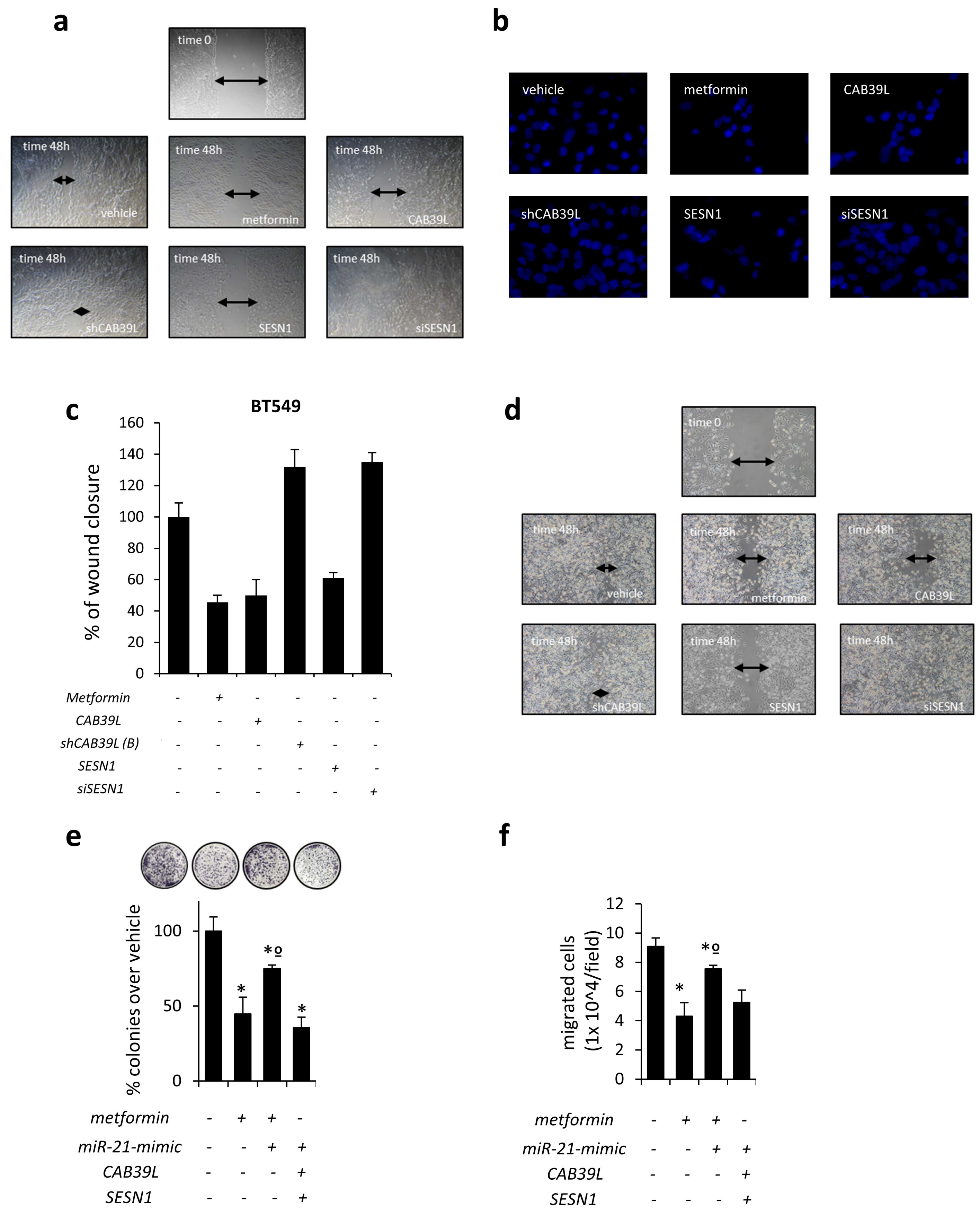

Supplementary Figure S5

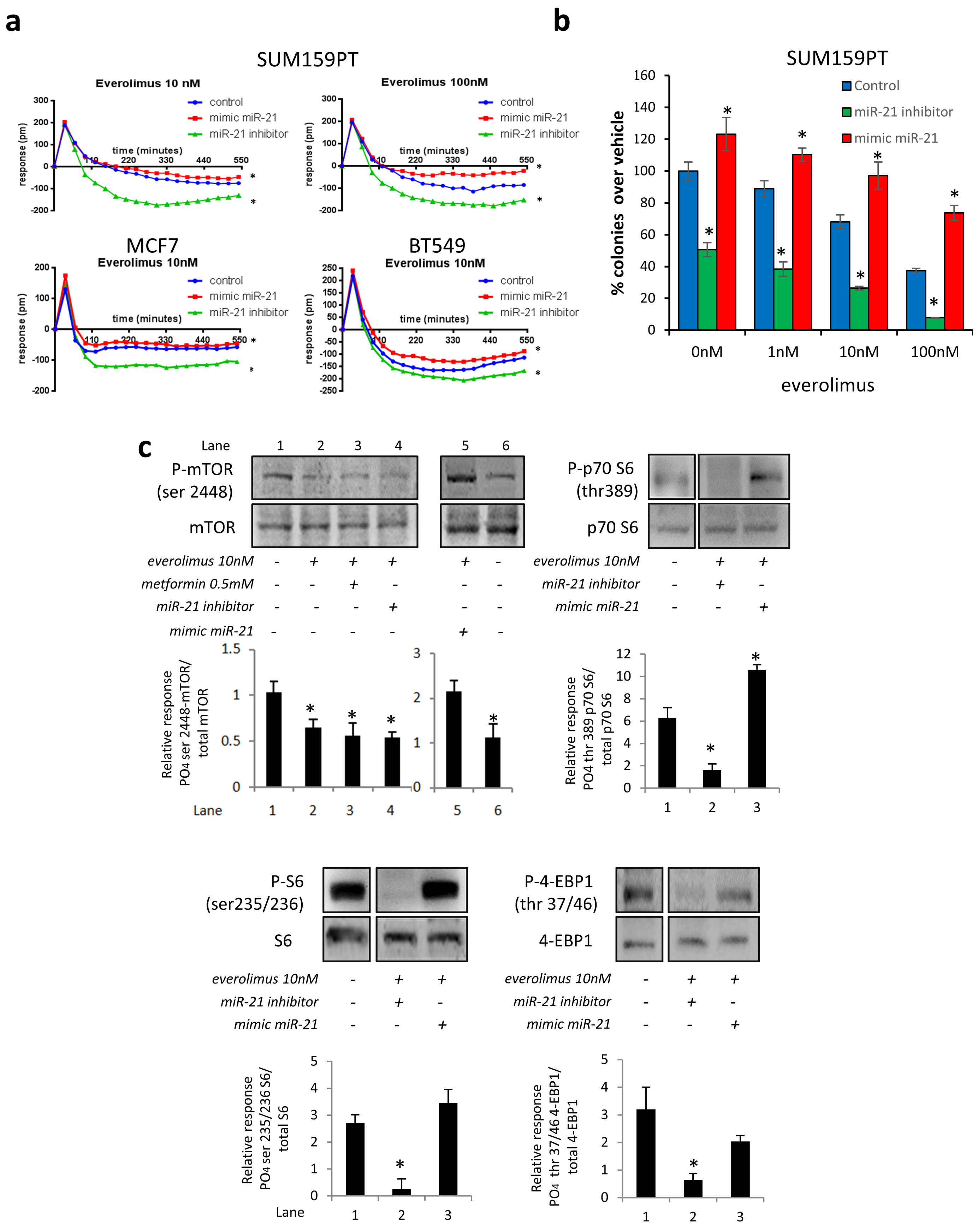

Supplement: Supplementary Information [file celldisc201722-s1.pdf]
